# Supplementary material for: A synthetic tubular molecular transport system
Source: Nat Commun. 2021 Jul 20;12:4393. doi: 10.1038/s41467-021-24675-8 (PMC8292359; doi:10.1038/s41467-021-24675-8)
Supplement: Supplementary file 1 — Supplementary Information [file 41467_2021_24675_MOESM1_ESM.pdf]

A synthetic tubular molecular transport system

Pierre Stömmer[1], Henrik Kiefer[2], Enzo Kopperger[3], Maximilian N. Honemann[1], Massimo Kube[1], Friedrich C. Simmel[3], Roland R. Netz[2], Hendrik Dietz[1]

[1]Lehrstuhl für Biomolekulare Nanotechnologie, Physik Department, Technische Universität München, Garching near Munich, Germany

[2]Lehrstuhl für Theoretische Bio- und Softmatter Physik, Physik Department, Freie Universität Berlin, Berlin, Germany

[3]Lehrstuhl für Physik Synthetischer Biosysteme, Physik Department, Technische Universität München, Garching near Munich, Germany

correspondence to dietz@tum.de

Supplementary Information

Contents:

Supplementary Figures 1-41
Supplementary Note 1

Supplementary Figures

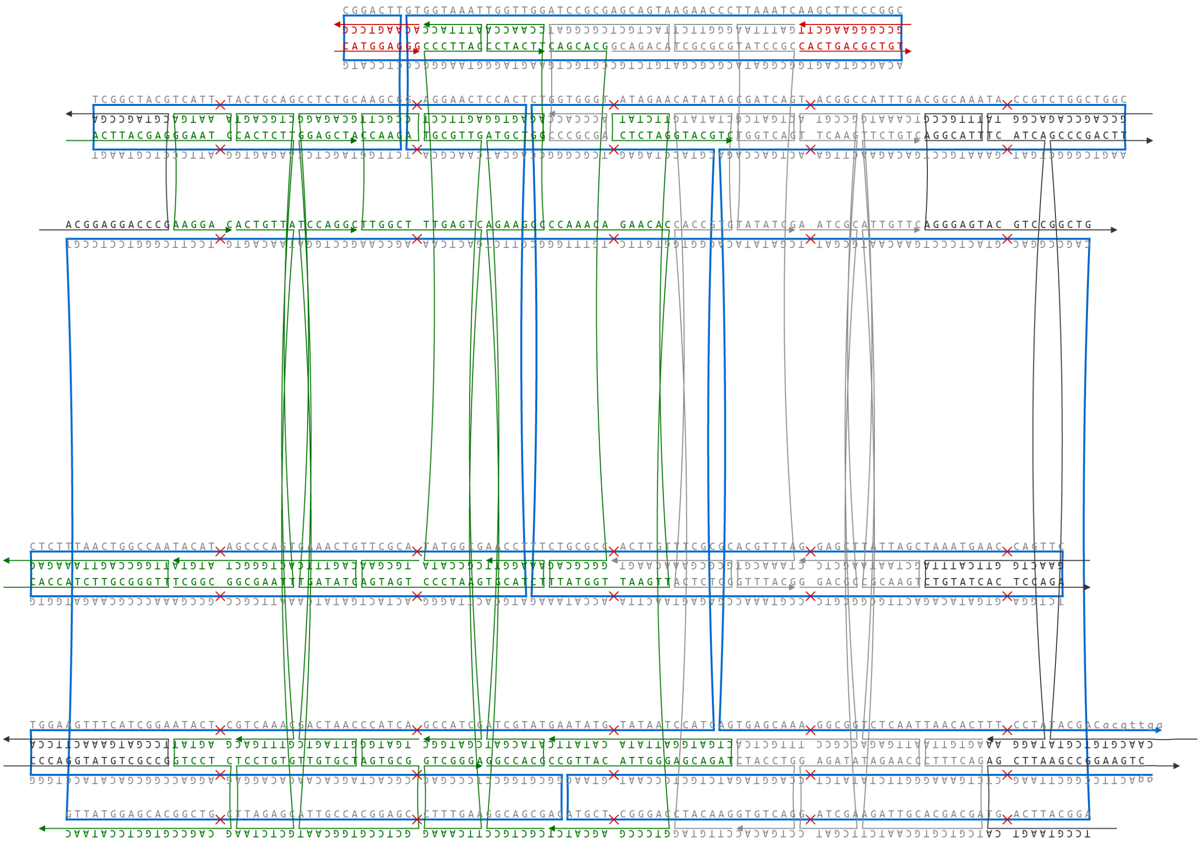

Supplementary Figure 1 | Strand diagram of the piston. Made with caDNAno v0.2 (*1*).

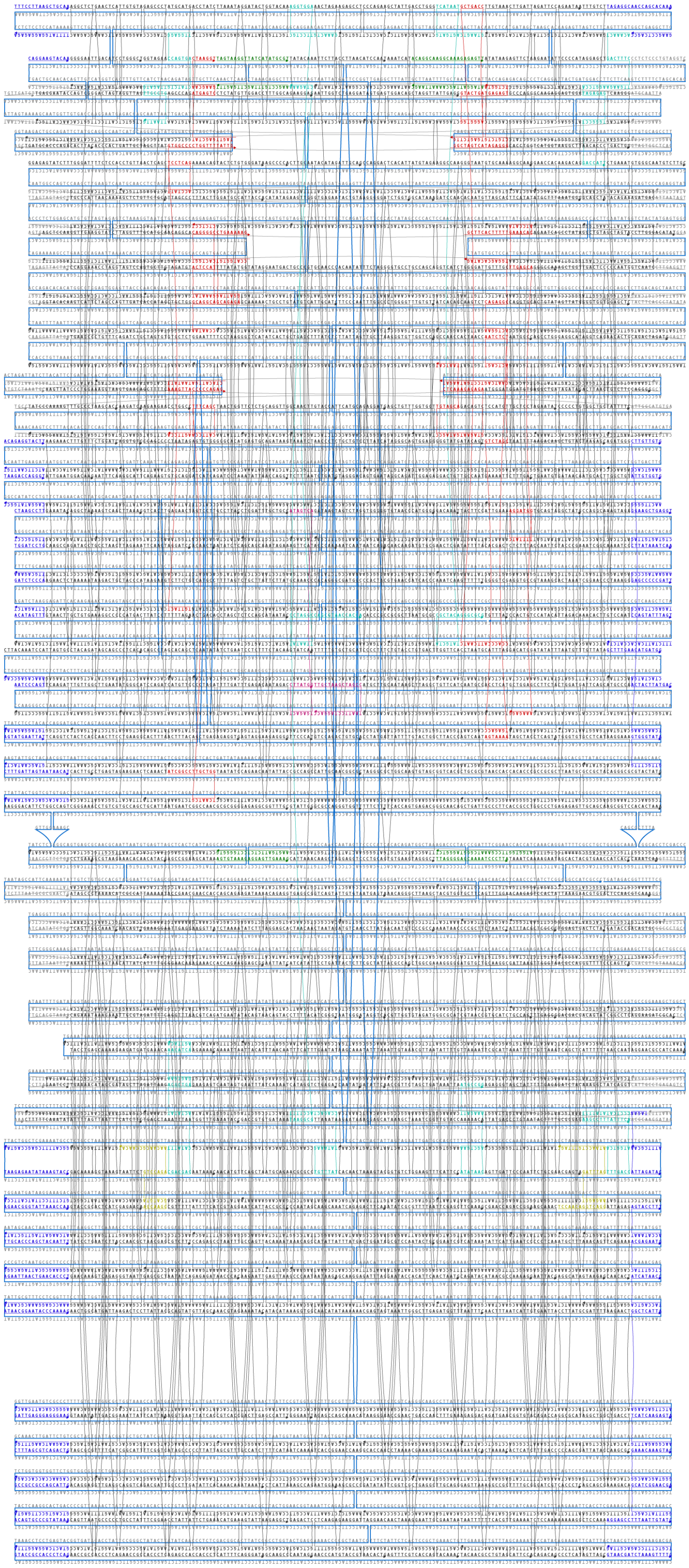

Supplementary Figure 2 | Strand diagram of the barrel. Made with caDNAno v0.2 (*1*).

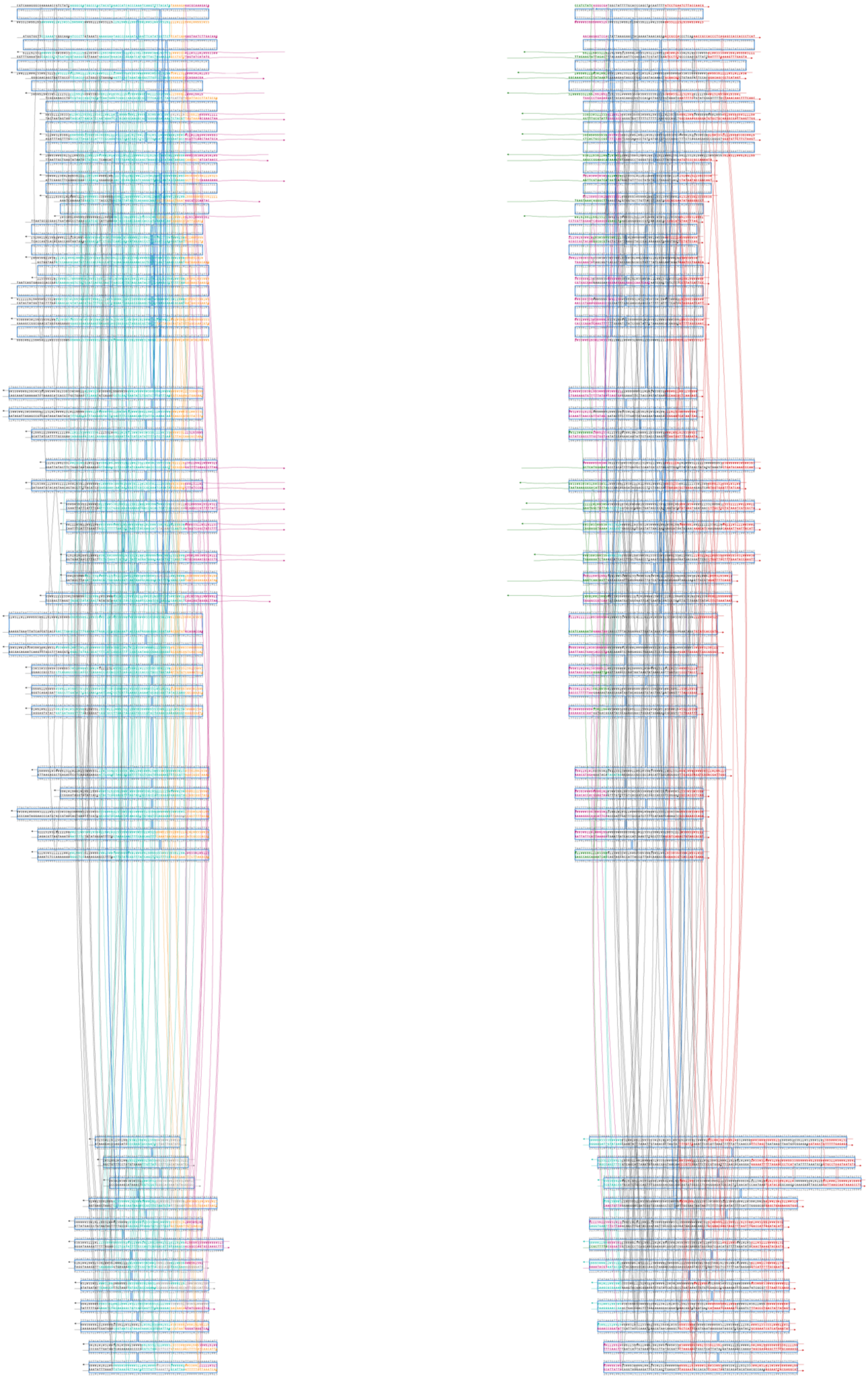

Supplementary Figure 3 | Strand diagrams of the cap building blocks (left: cap 1, right: cap 2). Made with caDNAno v0.2 (*1*).

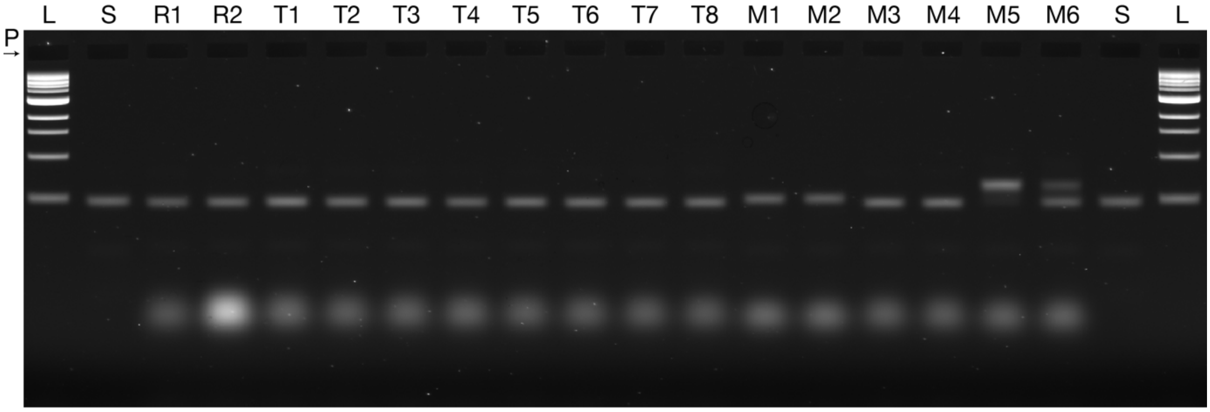

Supplementary Figure 4 | Piston folding screen. Laser-scanned image of a 2.5% agarose gel with 5.5 mM $MgCl_2$ run on a water bath at 90 V for 90 min on which the following samples were electrophoresed: L, 1kb ladder; S, 1033 bases long scaffold; R1, folding interval 60-44°C, 20 mM $MgCl_2$, 5x staple-to-scaffold excess; R2, 60-44°C, 20 mM $MgCl_2$, 10x staple-to-scaffold excess; lanes T1-T8: 20 mM $MgCl_2$; T1, folding interval 50-47°C; T2, folding interval 52-49°C; T3, folding interval 54-51°C; T4, folding interval 56-53°C; T5, folding interval 58-55°C; T6, folding interval 60-57°C; T7, folding interval 62-59°C; T8, folding interval 64-61°C; lanes M1-M6, folding interval 60-44°; M1, 5 mM $MgCl_2$; M2, 10 mM $MgCl_2$; M3, 15 mM $MgCl_2$; M4, 20 mM $MgCl_2$; M5, 25 mM $MgCl_2$; M6, 30 mM $MgCl_2$. P indicates the pockets. This exact gel-electrophoretic analysis was conducted once.

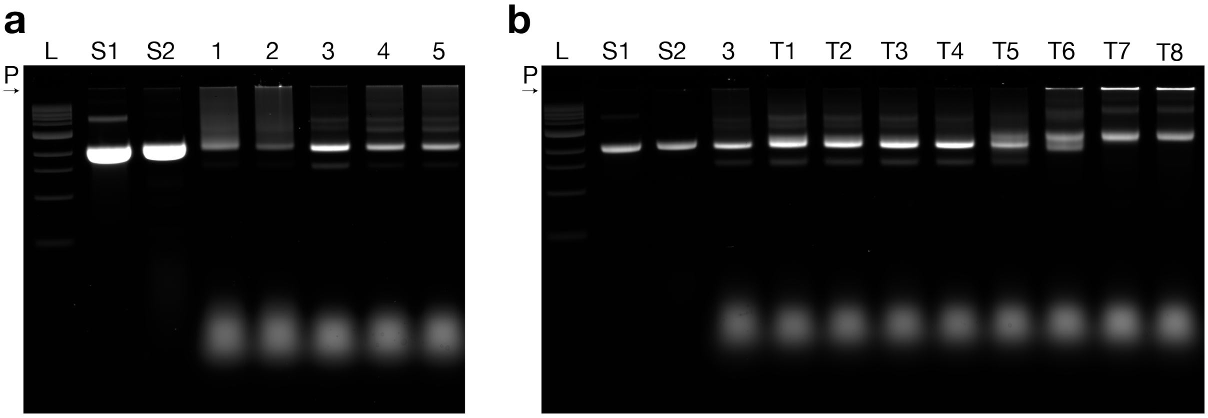

Supplementary Figure 5 | Barrel folding screens. Laser-scanned image of 2 **(a, b)** 1.5% agarose gels with 5.5 mM $MgCl_2$ run on a water bath at 90 V for 90 min on which the following samples were electrophoresed: L, 1kb ladder; S1, 7560 bases long scaffold type 1; S2, 7560 bases long scaffold type 2 (orthogonal to S1); 1-5 and T1-T8, barrel folding reactions, all starting with 15 min at 65°C; 1, 20 mM $MgCl_2$, 60-44°C 1 hour/°C; 2, 20 mM $MgCl_2$, 60-44°C 2 hours /°C; 3, 15 mM $MgCl_2$, 60-44°C 3 hours/°C; 4, 20 mM $MgCl_2$, 60-44°C 3 hours/°C; 5, 25 mM $MgCl_2$, 60-44°C 3 hours/°C; T1-T8: 15 mM $MgCl_2$, 3 hours/°C; T1, 50-47°C; T2, 52-49°C; T3, 54-51°C; T4, 56-53°C; T5, 58-55°C; T6, 60-57°C; T7, 62-59°C; T8, 64-61°C. P indicates the pockets. These exact gel-electrophoretic analyses were conducted once.

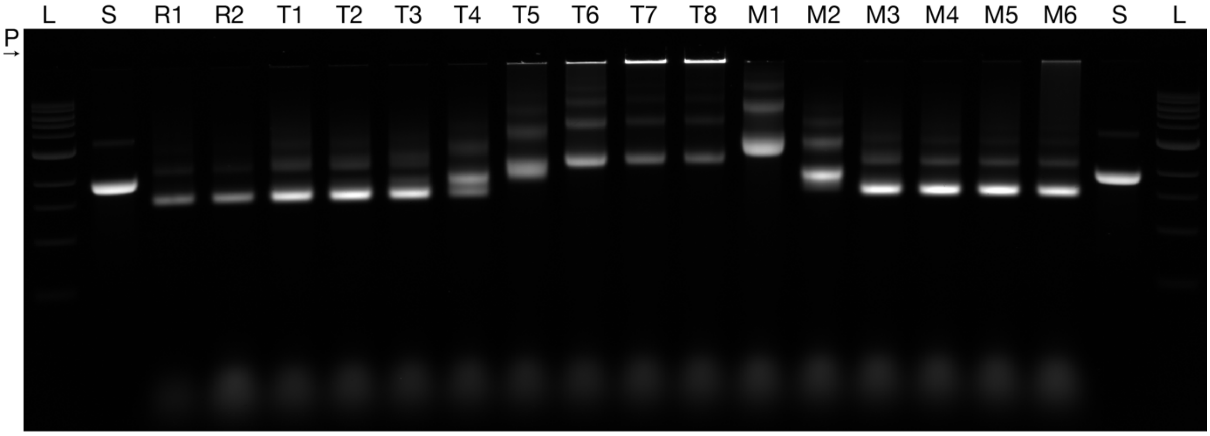

Supplementary Figure 6 | Cap1 folding screen. Laser-scanned image of a 2% agarose gel with 5.5 mM $MgCl_2$ run on a water bath at 90 V for 90 min on which the following samples were electrophoresed: L, 1kb ladder; S, 7560 bases long scaffold; R1, folding interval 60-44°C, 20 mM $MgCl_2$, 5x staple-to-scaffold excess; R2, 60-44°C, 20 mM $MgCl_2$, 10x staple-to-scaffold excess; lanes T1-T8: 20 mM $MgCl_2$; T1, folding interval 50-47°C; T2, folding interval 52-49°C; T3, folding interval 54-51°C; T4, folding interval 56-53°C; T5, folding interval 58-55°C; T6, folding interval 60-57°C; T7, folding interval 62-59°C; T8, folding interval 64-61°C; lanes M1-M6, folding interval 60-44°; M1, 5 mM $MgCl_2$; M2, 10 mM $MgCl_2$; M3, 15 mM $MgCl_2$; M4, 20 mM $MgCl_2$; M5, 25 mM $MgCl_2$; M6, 30 mM $MgCl_2$. P indicates the pockets. This exact gel-electrophoretic analysis was conducted once.

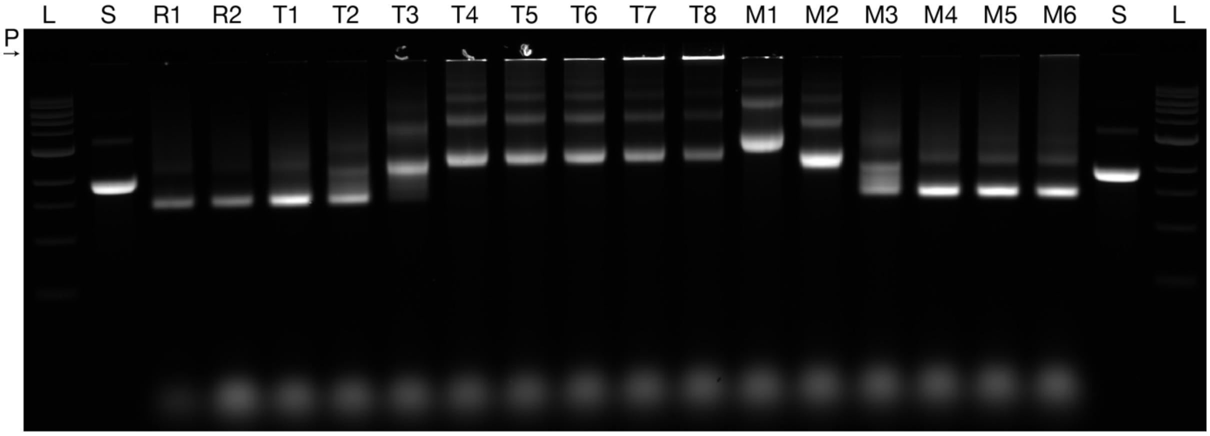

Supplementary Figure 7 | Cap2 folding screen. Laser-scanned image of a 2% agarose gel with 5.5 mM $MgCl_2$ run on a water bath at 90 V for 90 min on which the following samples were electrophoresed: L, 1kb ladder; S, 7560 bases long scaffold; R1, folding interval 60-44°C, 20 mM $MgCl_2$, 5x staple-to-scaffold excess; R2, 60-44°C, 20 mM $MgCl_2$, 10x staple-to-scaffold excess; lanes T1-T8: 20 mM $MgCl_2$; T1, folding interval 50-47°C; T2, folding interval 52-49°C; T3, folding interval 54-51°C; T4, folding interval 56-53°C; T5, folding interval 58-55°C; T6, folding interval 60-57°C; T7, folding interval 62-59°C; T8, folding interval 64-61°C; lanes M1-M6, folding interval 60-44°; M1, 5 mM $MgCl_2$; M2, 10 mM $MgCl_2$; M3, 15 mM $MgCl_2$; M4, 20 mM $MgCl_2$; M5, 25 mM $MgCl_2$; M6, 30 mM $MgCl_2$. P indicates the pockets. This exact gel-electrophoretic analysis was conducted once.

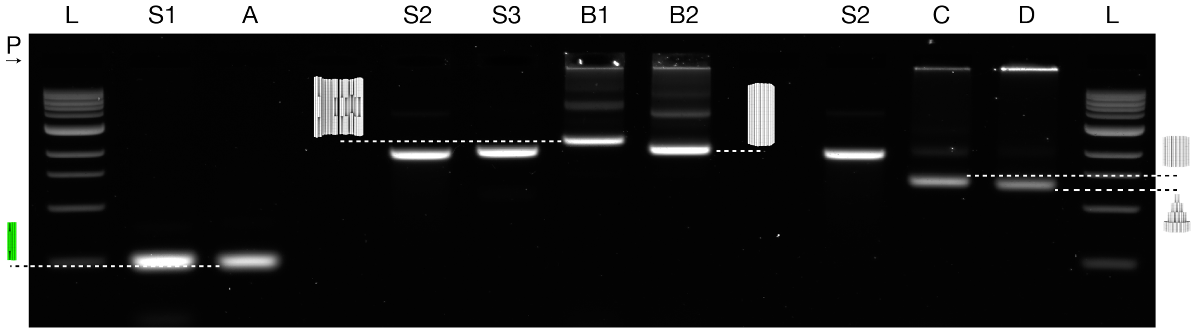

Supplementary Figure 8 | EMA quality control of monomers. Laser-scanned image (split into 3 images) of a 2% agarose gel with 5.5 mM $MgCl_2$ run on a water bath at 90 V for 90 min on which the following samples were electrophoresed: L, 1kb ladder; S1, 1033 bases long scaffold; A, folded and purified piston; S2, 7560 bases long scaffold type 1; S3, 7560 bases long scaffold type 2 (orthogonal to S1); B1, folded and purified barrel in a dynamic/open configuration; B2, folded and purified barrel in a permanently closed configuration; C, folded and purified cap 1; D, folded and purified cap 2. Dashed lines point from the corresponding bands to models of the folded and purified structures. P indicates the pockets. The total number of similarly conducted gel-electrophoretic analyses of samples prepared following the same protocol as illustrated was 3 times and showed similar results. This exact gel-electrophoretic analysis was conducted once.

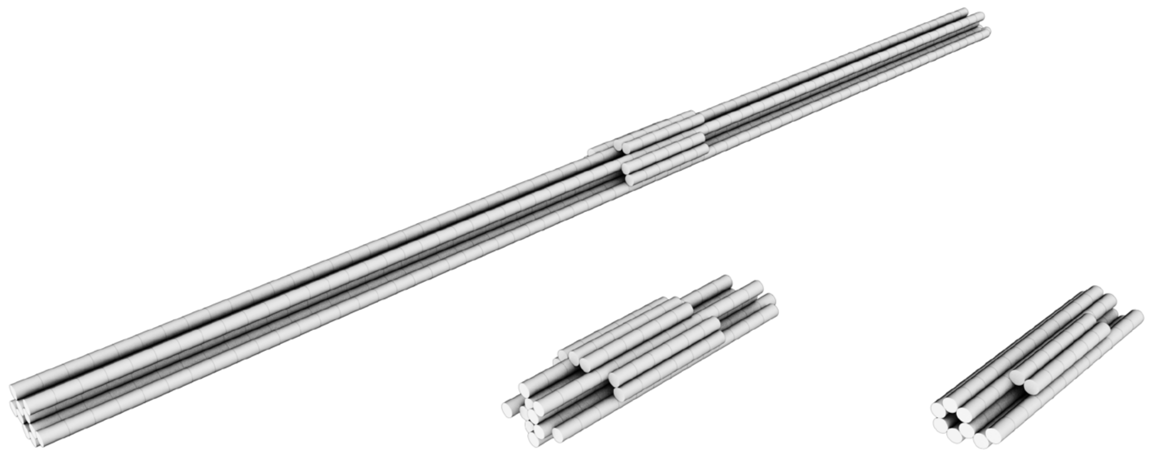

Supplementary Figure 9 | Different piston variants. Models of piston variants. **Left:** variant 1 (250 x 12 x 12 nm). **Middle:** variant 2 (40 x 12 x 12 nm). **Right:** variant 3 (40 x 8 x 12 nm). Cylinders represent DNA double- helices.

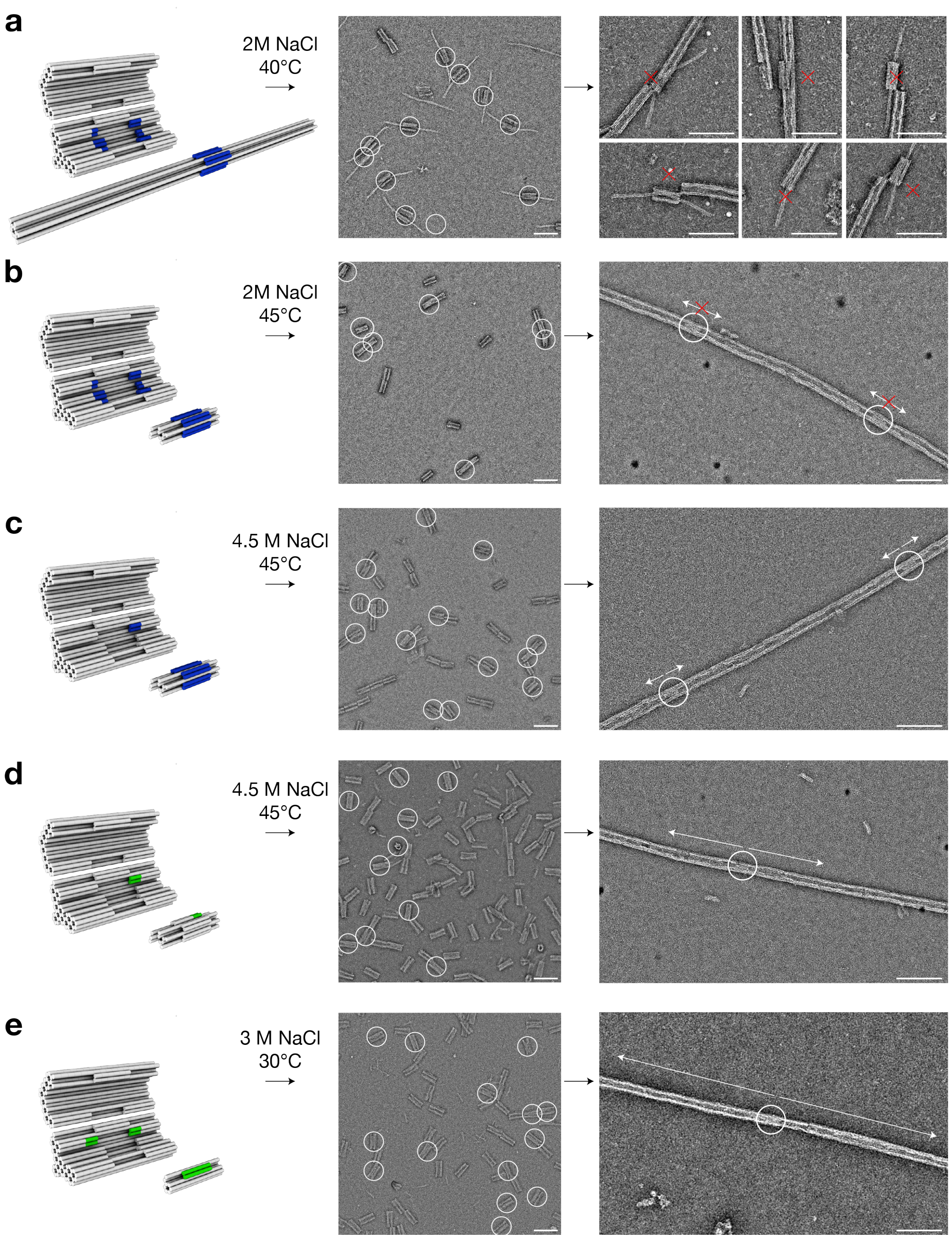

Supplementary Figure 10 | Loading the tunnels with different piston variants. (a-e) Left: models of the barrel and piston variants. Cylinders represent DNA double-helices. The barrel is illustrated in an open conformation. Blue and green protrusions and recesses on the barrel and pistons highlight interaction sites. Blue: blunt-end stacking interactions; green: hybridization of single stranded overhangs and single stranded scaffold loops on the piston and the barrel binding site. **Middle:** typical

field of view TEM micrographs of assembled barrel-piston dimers imaged with a Philips CM100 microscope. White circles highlight correctly assembled dimers. **Right:** typical field of view TEM micrographs of extended tracks containing loaded barrels imaged with a Philips CM100 and an FEI Tecnai120 microscope. White circles highlight pistons inside correctly formed filaments. Red crosses highlight assembly defects that inhibit piston movement. **(a)** Piston variant 1, folded from a 7560 bases long scaffold, is approximately 4 times longer than the barrel in the helical direction. It binds to the barrel binding site by blunt-end stacking interactions. This reaction occurs at 40°C and 2 M NaCl (left). Negative-staining TEM confirms correctly assembled barrel-piston dimers (middle). The filament assembly step leads to defects with the piston often sticking out laterally at barrel-barrel interfaces. We therefore tested a second piston variant **(b)** which is shorter than the barrel and was folded from a 1545 bases long scaffold. Piston variant B was docked to the barrel via blunt-end stacking interactions. This reaction occurs at 45°C and 2 M NaCl (left). Negative-staining TEM confirms correctly assembled barrel-piston dimers (middle). The filament assembly now results in correctly enclosed pistons inside of filaments (right). However, even at very low ionic strengths (0.3-0.5 M NaCl) the piston would not move, which we attributed to failure to undock from the binding site. For variant **(c)**, we reduced the number of blunt-end stacking bonds from 12 down to 2. The other blunt ends were passivated with 5 T overhangs. The dimerization reaction now occurs at 45°C and 4.5 M NaCl (left). Negative-staining TEM confirms correctly assembled barrel-piston dimers (middle). The filament assembly shows correctly enclosed pistons inside of filaments (right). However, real-time fluorescence mobility experiments at low ionic strengths (0.3-0.5 M NaCl) again showed very low mobility of this piston variant. Further reducing the NaCl concentration led to filament disassembly. Reducing the blunt-end stacking interaction between the barrel and piston even further was not practical, since the loading reaction ceased to work. For variant **(d)**, the docking site of the piston now features single stranded scaffold loops whose sequences are complementary to single stranded overhangs in the barrel binding site. The dimerization reaction occurs at 45°C and 4.5 M NaCl (left). Negative-staining TEM confirms correctly assembled barrel-piston dimers (middle). The filament assembly again resulted in correctly enclosed pistons inside of filaments (right). Mobility experiments were performed upon adding invader strands that bind to the piston, thereby releasing it from its initial binding site by toehold-mediated strand displacement. This piston variant showed improved mobility compared to the previous variant (c). However, it still only covered total displacements of up to several 100 nm. For variant **(e)**, we reduced the cross section of the piston to make its motion less susceptible to potential constrictions in the track. Variant E features single stranded scaffold loops at both ends of the docking site. The sequences are complementary to single stranded overhangs at the barrel binding site. The dimerization reaction occurs at 30°C and 3 M NaCl (left). The filament assembly resulted in correctly enclosed pistons inside of filaments (right). Mobility experiments were performed upon adding invader strands that bind to the piston, thereby releasing it from its initial binding site by toehold-mediated strand displacement. Piston variant e showed the highest diffusivity and the longest travel range along the track. All TEM micrographs were high-pass filtered (radius: 25 pixels). All scale bars: 100 nm. The

total number of similarly conducted TEM analyses of samples prepared following the same protocol as illustrated here was 2 times for all conditions shown here and showed similar results.

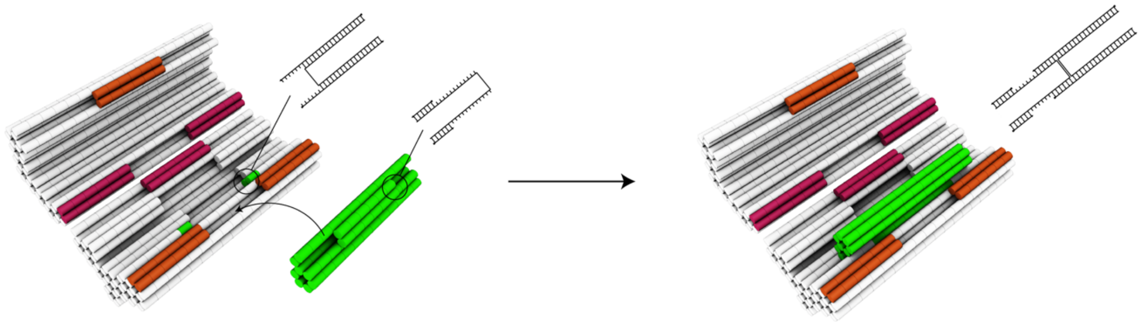

Supplementary Figure 11 | Details of the barrel-piston loading reaction. Models of the barrel (white) and the piston (green) objects. Cylinders indicate DNA double-helices. Orange and magenta cylinders highlight protrusions and recesses involved in closing the barrel. Green cylinders on the inside wall of the barrel object highlights the binding spot for the piston object. Black straight lines pointing away from the black circles highlight zoom-ins on a recess of the barrel (binding spot) and a protrusion on the piston. Zoom-ins: Long black lines indicate the backbone of DNA; shorter black lines indicate bases of DNA. The binding spot on the barrel consists of overall 2 x 6 and 2 x 4 bases long single-stranded overhangs, two on each side (here only two are highlighted on one side). The zoom-in on the piston object highlights the single-stranded scaffold loop at one end of the protrusion (here only one side is highlighted, the other side of the protrusion follows the same design principle).

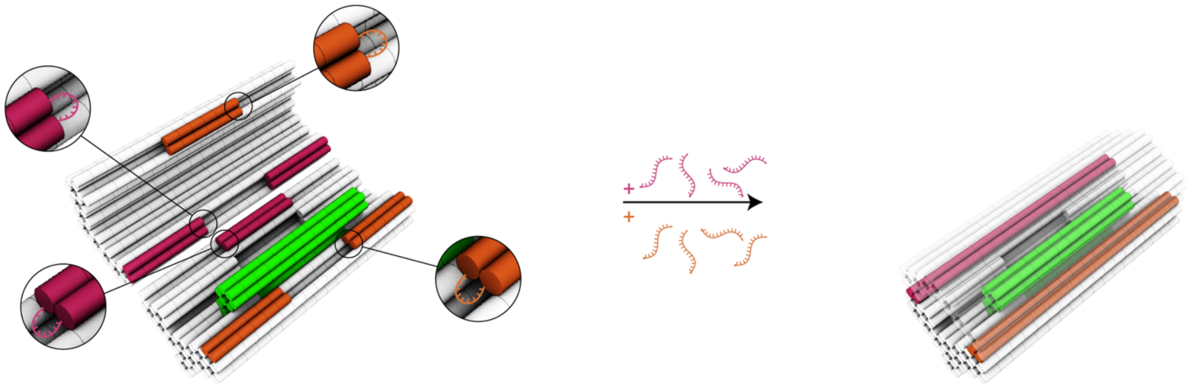

Supplementary Figure 12 | Barrel closing mechanism. Left: Model of a barrel (white)-piston (green) dimer object in an open confirmation. The ends of the orange and magenta protrusions and recesses are initially left single-stranded (insets). **Right:** Oligonucleotides are added in solution and hybridize to the orange and magenta single-stranded loops, thus fixing the barrel in a closed conformation.

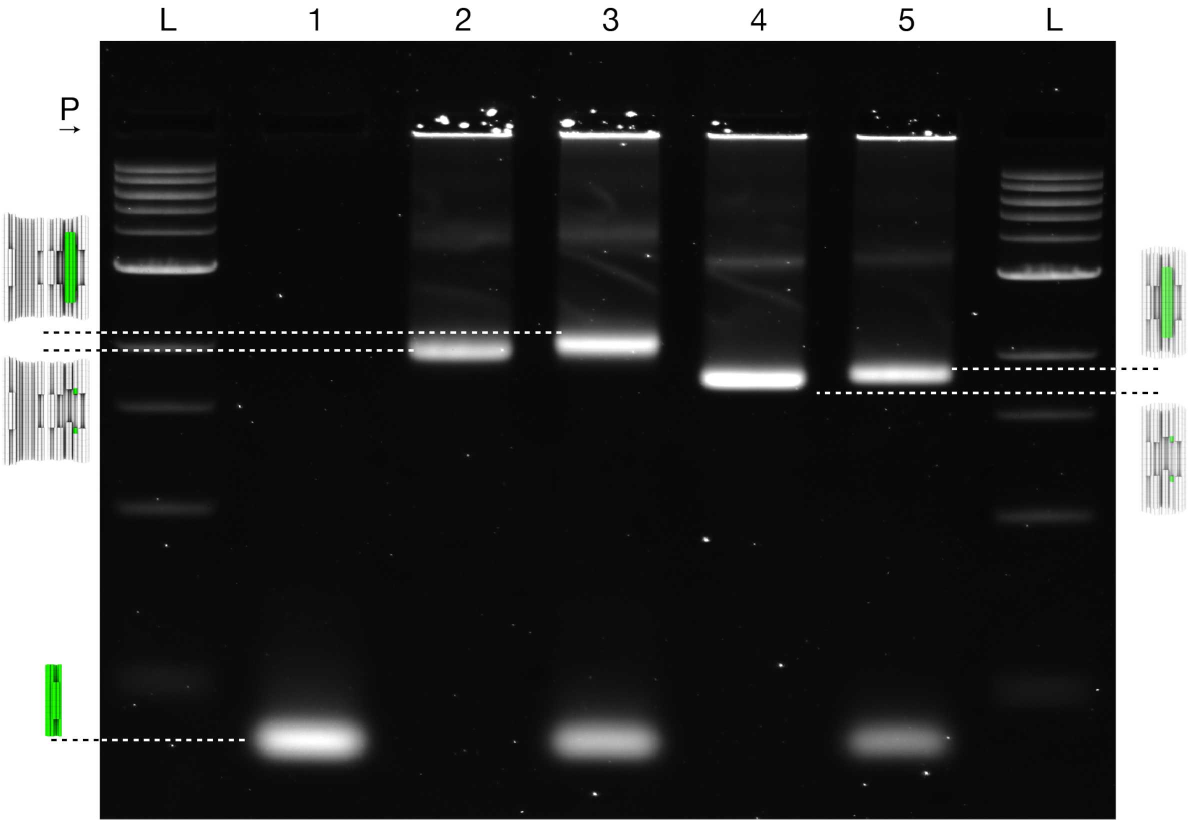

Supplementary Figure 13 | EMA quality control of barrel-piston dimers. Laser-scanned image of a 2.5% agarose gel with 21 mM $MgCl_2$ run on an ice-water bath at 70 V for 180 min on which the following samples were electrophoresed: 1, piston; 2, open barrel; 3, open piston-barrel dimer; 4, closed barrel; 5, closed piston-barrel dimer. Dashed lines point from the corresponding bands to models of the assembled structures. The total number of similarly conducted gel-electrophoretic analyses of samples prepared following the same protocol as illustrated here was 3 times and showed similar results. This exact gel-electrophoretic analysis was conducted once.

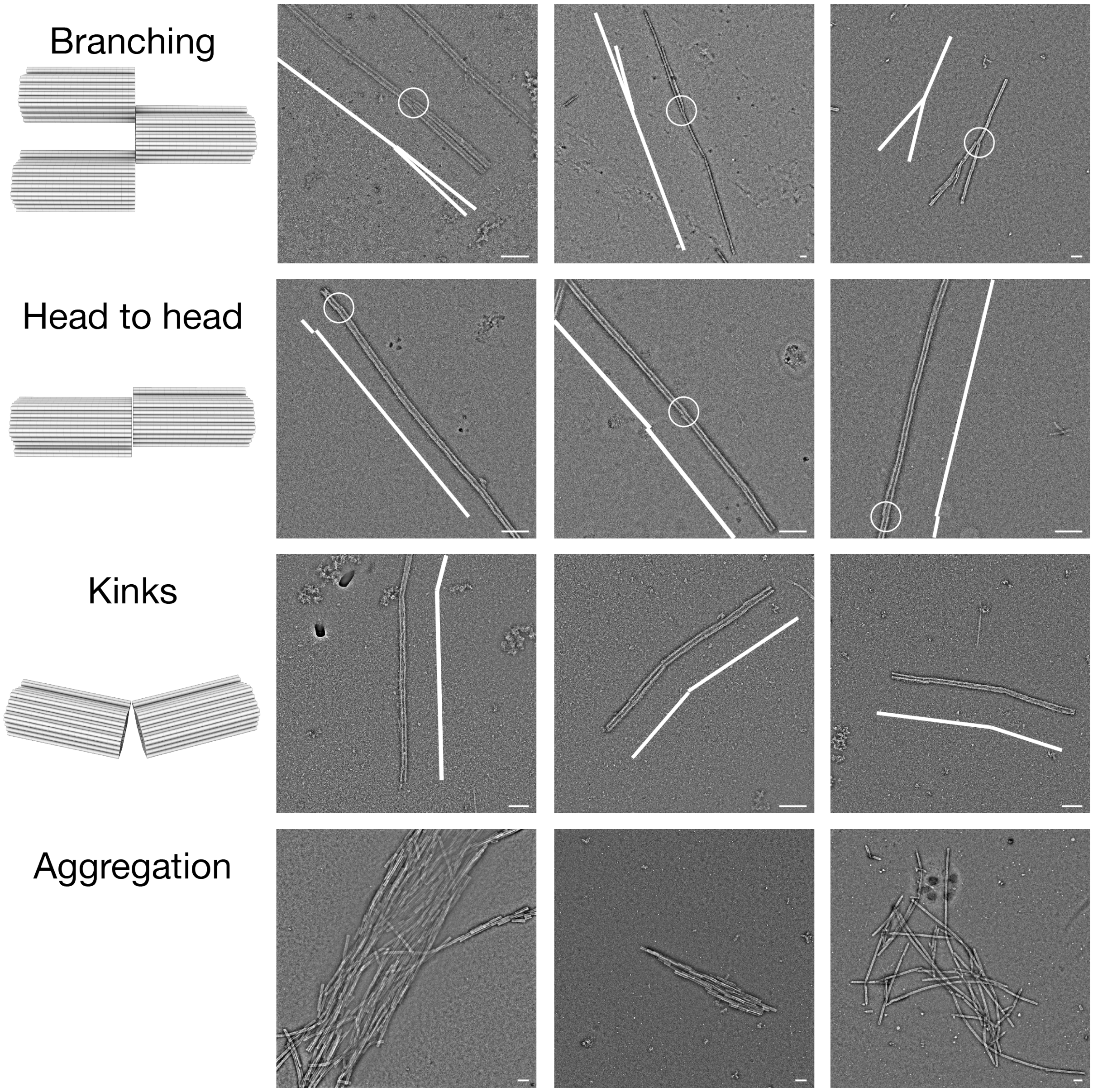

Supplementary Figure 14 | Filament defect types. Left: models of barrels that are attached to each other in wrong orientations. **Right:** exemplary negative-staining TEM images of filaments with defects that represent roadblocks to piston movement. To reduce the occurrence of branching we use two types of polymerization oligonucleotides, one which generate sticky end overhangs and another which produce blunt-ended stacking sites. Sticky end oligonucleotides partly bind to scaffold loops on one end of a barrel and partly on the opposite end of another barrel, thereby bridging two barrels. Stacking end oligonucleotides only bind to scaffold loops on one end of a barrel, resulting in a blunt ended interface. Sticky ends were placed on all helices of the inner layer of the barrel, stacking ends were placed on all helices of the outer layer of the barrel. To reduce head-to-head or tail-to-tail polymerization we implemented a two-step polymerization procedure (Supplementary Figure15). Occurrence of kinks could be reduced by using a polymerization oligonucleotide to barrel stoichiometry of no greater than 4:1. The tendency to form aggregates was least when polymerization was performed in the presence of 2 M NaCl at 40°C out of all conditions tested. Using an additional

incubation step of 1 hour at 40°C and 2 M NaCl after closing assembled barrel-piston dimers and right before triggering the polymerization reaction by adding the polymerization oligonucleotides was also beneficial. Images were acquired with a Philips CM100 microscope. All TEM micrographs were high-pass filtered (radius: 25 pixels). All scale bars: 100 nm. The total number of similarly conducted TEM analyses of samples prepared following the same protocol as illustrated here was 2 times for all conditions shown here and showed similar results.

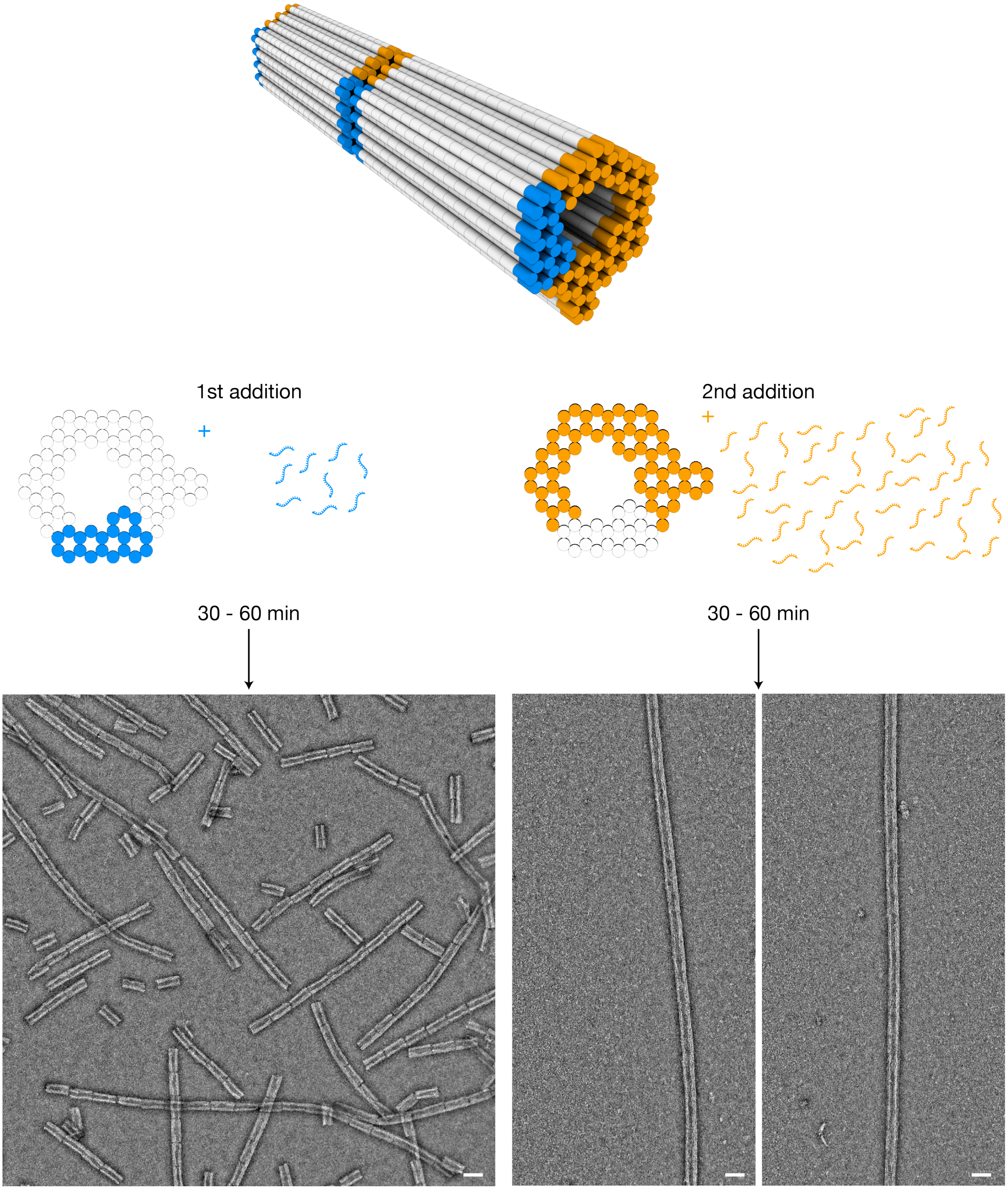

Supplementary Figure 15 | Best polymerization strategy. **Top:** models of 2 barrels. White, blue and orange cylinders represent DNA double-helices. **Middle:** cross sectional view of the barrel. **Left:** first addition of oligonucleotides that serve as a first set of polymerization oligonucleotides. They bind to the scaffold loops at the end of 1 (blue circles) of 6 sides of the barrel. **Bottom left:** typical field of view negative-staining TEM micrograph of barrels 30-60 min after adding the first set of polymerization oligonucleotides and incubating at 40°C and 2 M NaCl, imaged with a Philips CM100 microscope. **Middle right:** second addition of oligonucleotides that serve as a second set of polymerization oligonucleotides. They bind to the scaffold loops at the end of 5 (orange circles) of 6 sides of the barrel.

Bottom right: typical field of view negative-staining TEM micrograph of barrels 30-60 min after adding the first set of polymerization oligonucleotides and incubating at 40°C and 2 M NaCl and then adding the second set of polymerization oligonucleotides and incubating at 40°C and 2 M NaCl, imaged with a Philips CM100 microscope. All TEM micrographs were high-pass filtered (radius: 25 pixels). Scale bars: 50 nm. The total number of similarly conducted TEM analyses of samples prepared following the same protocol as illustrated here was 3 times for both conditions shown here and showed similar results.

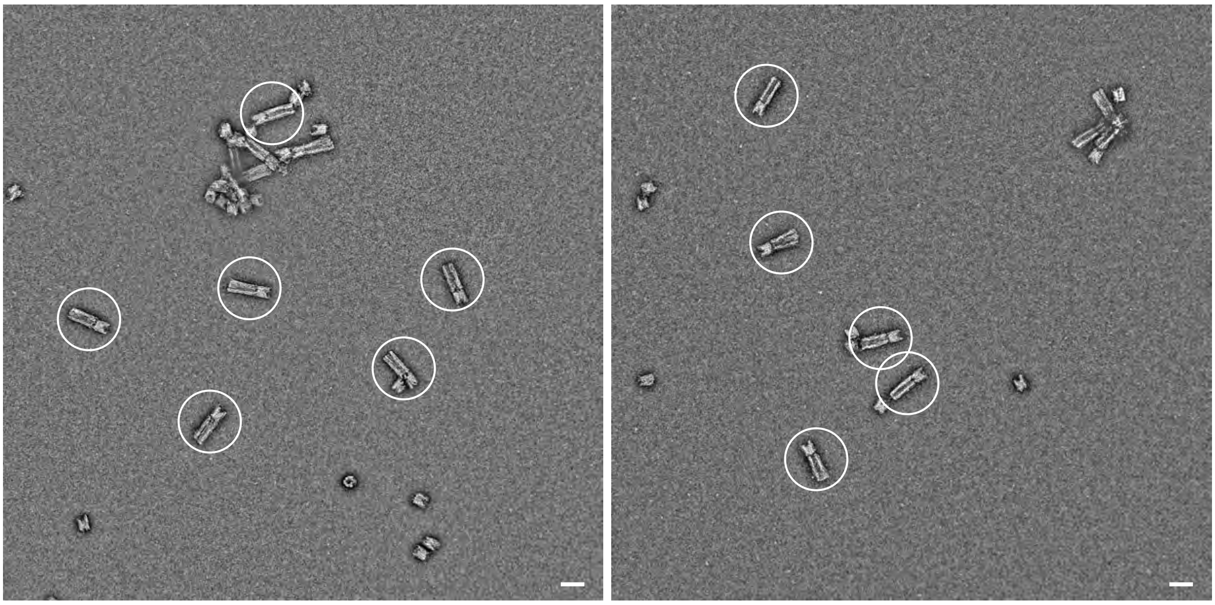

Supplementary Figure 16 | TEM quality control of cap1-barrel dimers. Typical field of view negative-staining TEM micrographs of assembled barrel-cap1 dimers, imaged with a Philips CM100 microscope. The TEM micrographs were high-pass filtered (radius: 25 pixels). Scale bars: 50 nm. The total number of similarly conducted TEM analyses of samples prepared following the same protocol as illustrated here (steps 10 and 11 in the transport system sample preparation protocol) was 3 times and showed similar results.

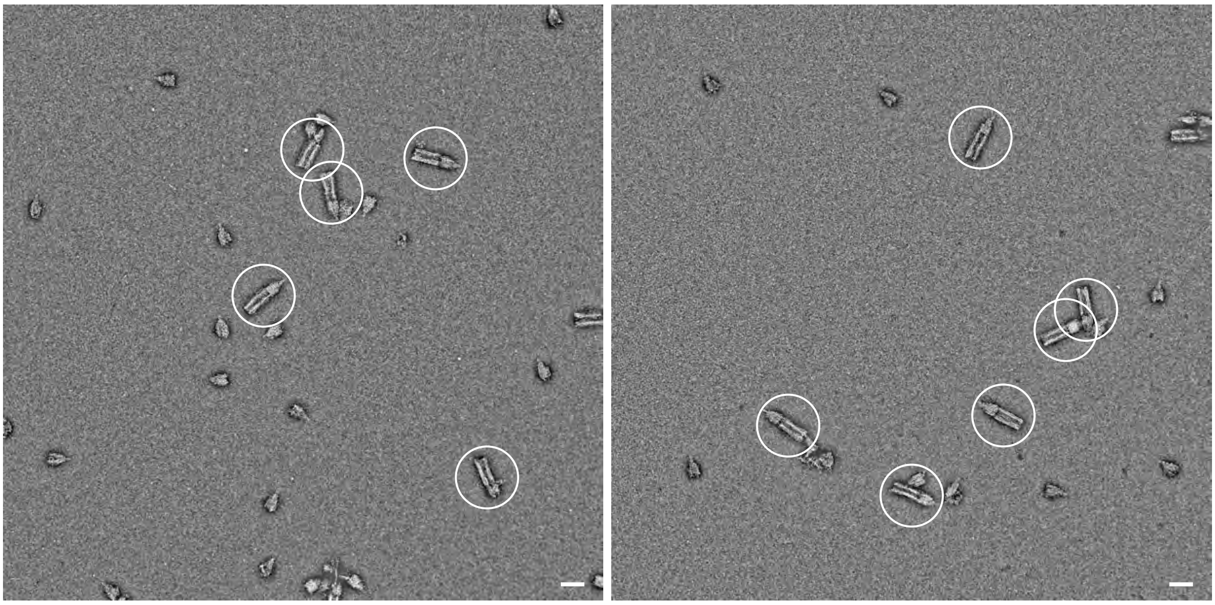

Supplementary Figure 17 | TEM quality control of cap2-barrel dimers. Typical field of view negative-staining TEM micrographs of assembled barrel-cap2 dimers, imaged with a Philips CM100 microscope. The TEM micrographs were high-pass filtered (radius: 25 pixels). Scale bars: 50 nm. The total number of similarly conducted TEM analyses of samples prepared following the same protocol as illustrated here (steps 10 and 11 in the transport system sample preparation protocol) was 3 times and showed similar results.

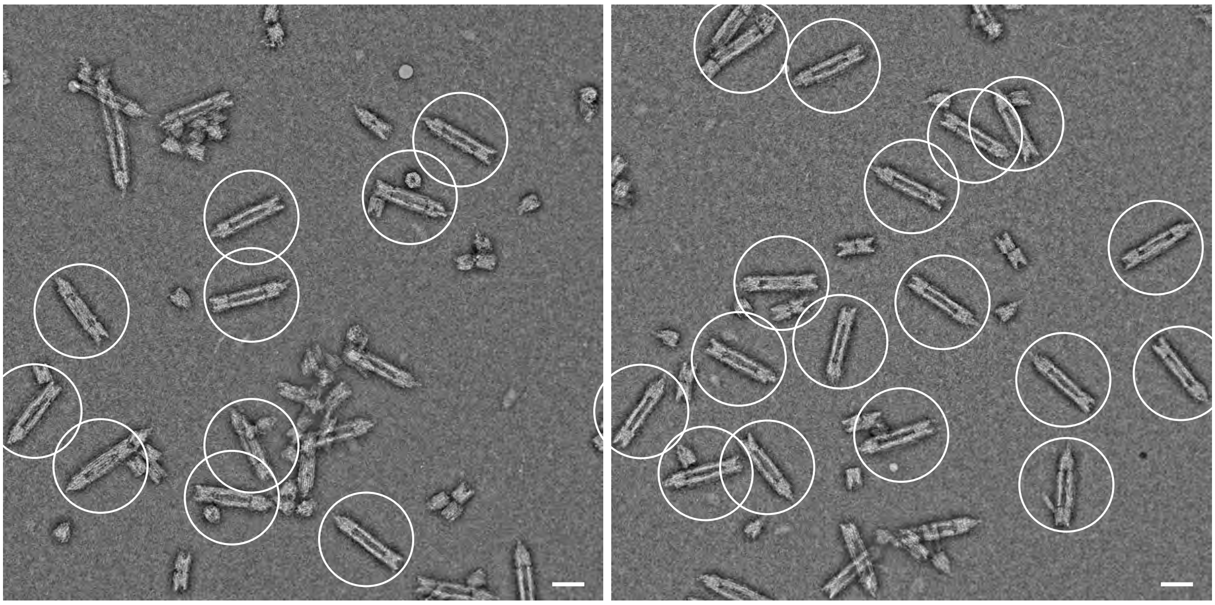

Supplementary Figure 18 | TEM quality control of cap1-barrel-cap2 trimers. Typical field of view negative-staining TEM micrographs of assembled cap1-barrel-cap2 trimers, imaged with a Philips CM100 microscope. The TEM micrographs were high-pass filtered (radius: 25 pixels). Scale bars: 50 nm. The total number of similarly conducted TEM analyses of samples prepared following the same protocol as illustrated here (steps 10 and 11 in the transport system sample preparation protocol) was 2 times and showed similar results.

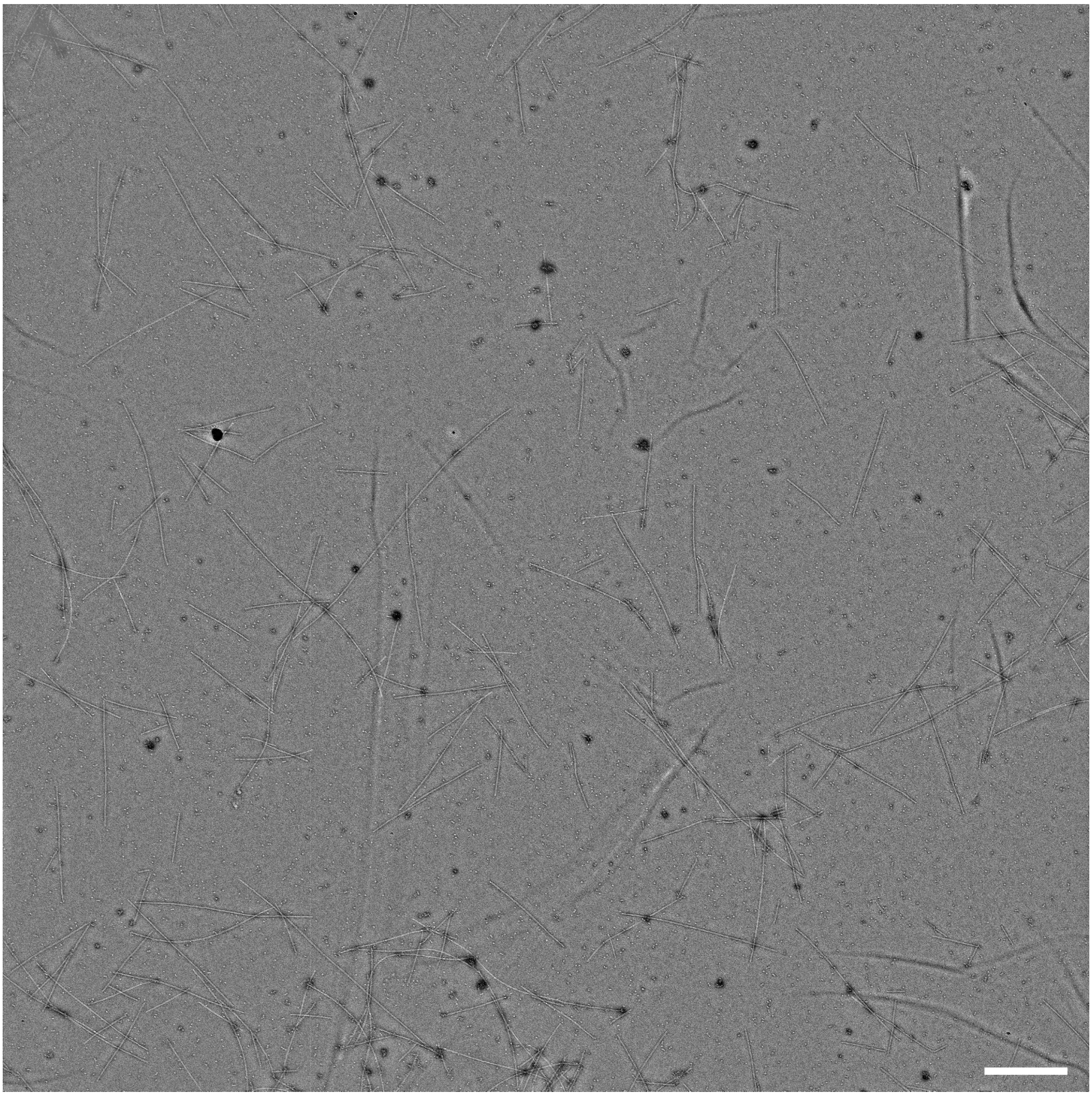

Supplementary Figure 19 | TEM quality control of assembled tracks containing pistons. Typical field of view negative-staining TEM micrograph of assembled transport systems imaged with a Jeol JEM3200 FSC microscope. The TEM micrograph was high-pass filtered (radius: 50 pixels). Scale bar: 1 µm. The total number of similarly conducted TEM analyses of samples prepared following the same protocol as illustrated in the transport system sample preparation protocol was 5 times and showed similar results.

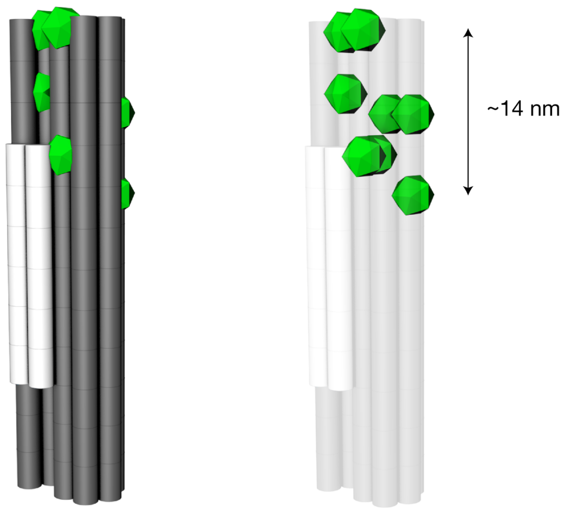

Supplementary Figure 20 | Fluorescent labeling of the piston. Illustration of the Cyanine-3 dye placement on the piston. Cylinders represent DNA double-helices. Green objects illustrate Cyanine-3 dyes. The 8 Cyanine-3 dyes are placed over a distance of 14 nm.

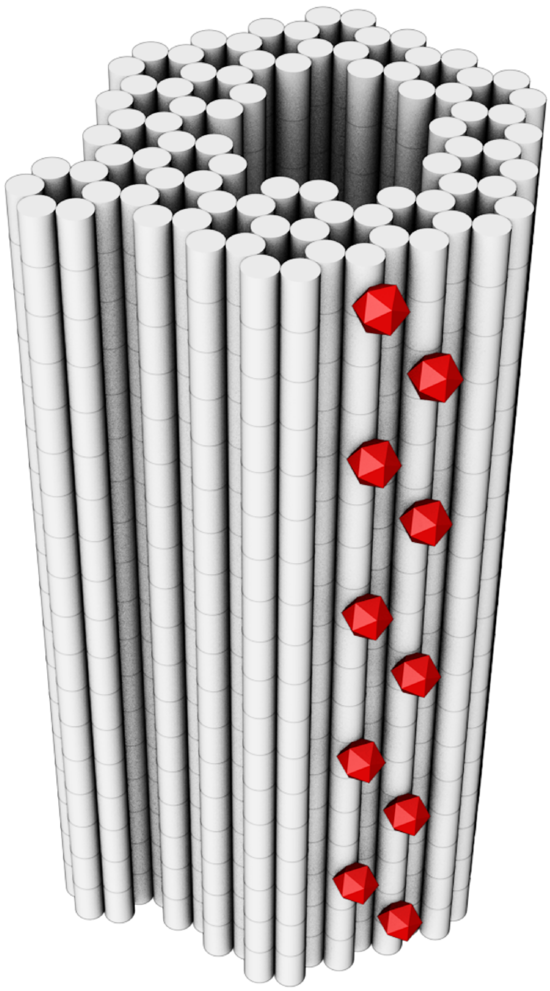

Supplementary Figure 21 | Fluorescent labelling of the barrel. Illustration of the Cyanine-5 dye placement on the barrel. Cylinders represent DNA double-helices. Red objects illustrate Cyanine-5 dyes.

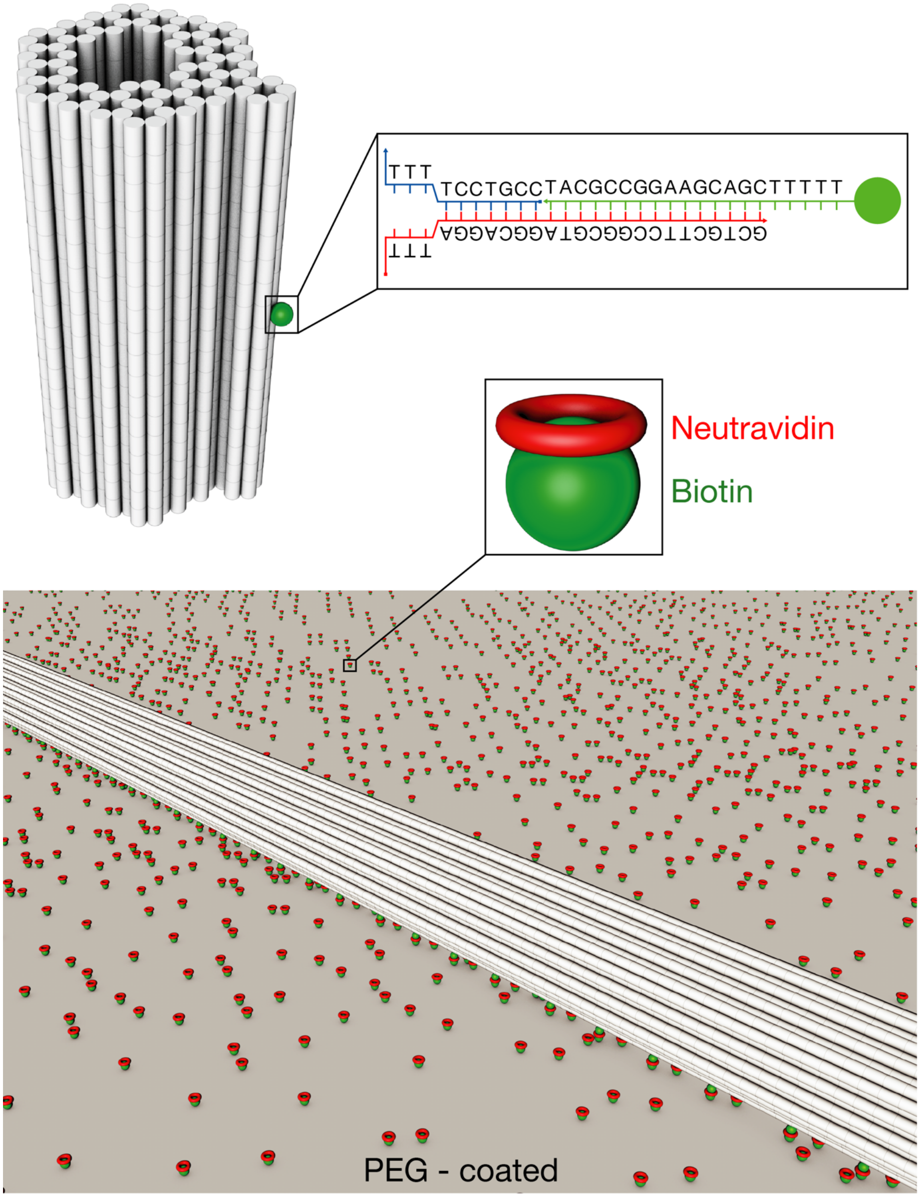

Supplementary Figure 22 | Biotin placement on the barrel. Illustration of the biotin placement on the barrel. **Top left:** Cylinders represent DNA double-helices. Green sphere represents biotin. **Top right:** Zoom-in on the biotin anchor. Letters indicate the sequences, straight lines represent single bases and backbones of three (red, blue, green) oligonucleotides. **Bottom:** Illustration of a tunnel bound to a pegylated glass surface via neutravidin. Grey surface represents the pegylated glass surface (protocol in materials & methods). White cylinders represent DNA double-helices. Green spheres represent biotin, red objects represent neutravidin. Zoom-in highlights a biotin on the glass surface, bound to one neutravidin.

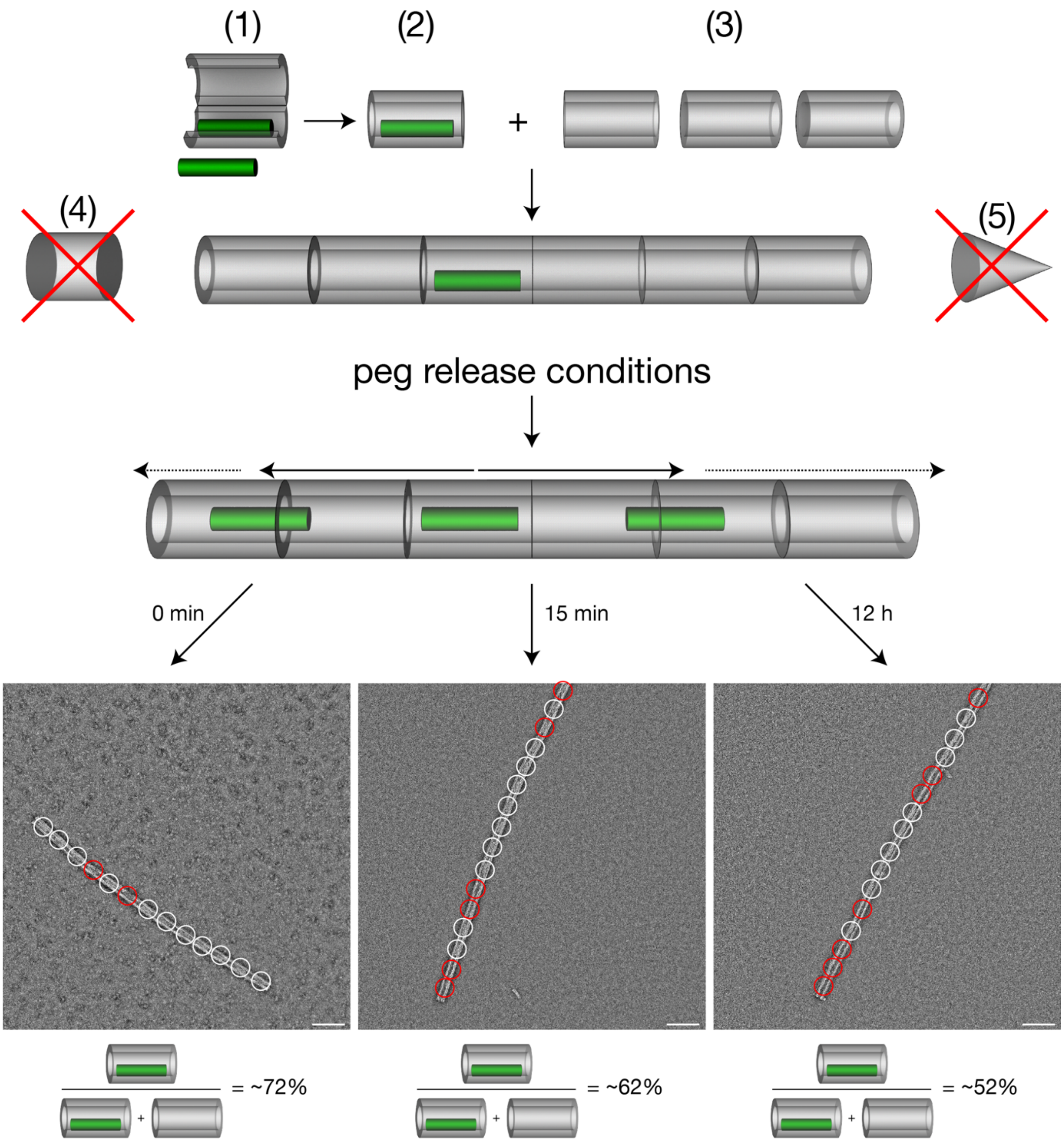

Supplementary Figure 23 | TEM "efflux" analysis of low mobility piston-track designs. Top: Schematic illustration of an experiment designed to test very low mobility pistons for their functionality. Grey cylinders represent the barrel, green objects represent the piston. For this experiment, filaments with pistons inside but without any caps attached at the ends are needed. To this end, the piston variant in question was bound to the barrel at barrel to piston ratios between 1:1 and 2:1 **(1)**. The barrel was then subsequently closed permanently **(2)**. Polymerization oligonucleotides were then added to this sample, containing barrel-piston dimers and empty barrels **(3)**. Steps **(4)** and **(5)**, adding both caps to close the filament ends, were omitted, resulting in open filaments, with different amounts of pistons inside, depending on the initial barrel to piston ratio. These open filament samples were then split into separate reaction tubes and subjected to different piston releasing conditions, based on the specific piston variant in question, and incubated at these conditions for various lengths of time. The resulting

samples were then analyzed using negative-staining TEM (bottom), by counting the number of vacant (highlighted by red circles) and occupied (highlighted by white circles) piston binding spots inside of filaments. **Bottom:** 3 exemplary negative-staining TEM micrographs of such samples at different points in time and the respective fraction of occupied binding spots and total number of binding spots (below TEM micrographs). The TEM micrographs were high-pass filtered (radius: 25 pixels). Scale bars: 100 nm. Several hundred binding spots were analyzed and counted for all samples. **Bottom left:** this specific sample was not subjected to piston releasing conditions and showed ~72% occupied binding spots. **Bottom middle:** this sample, initially from the same pool as the first sample (left), incubated at piston releasing conditions for 15 min before TEM grids were prepared. It shows ~62% occupied binding spots. **Bottom right:** this sample incubated at piston releasing conditions for 24 hours before TEM grids were prepared. It shows ~52% occupied binding spots.

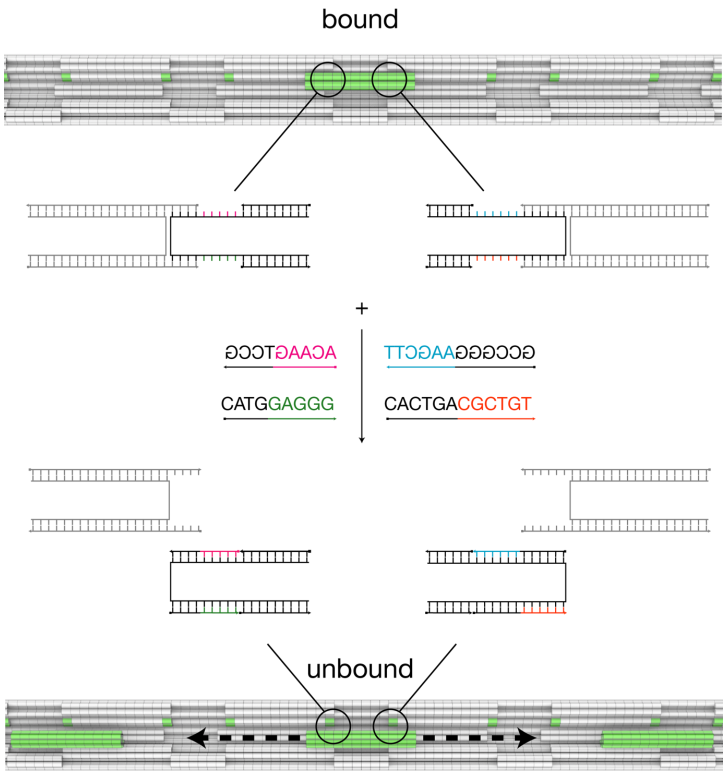

Supplementary Figure 24 | Illustration of the piston release mechanism. Cylinders represent DNA double-helices. **Top:** snippet of a filament with a piston (green object) trapped inside of the filament, bound in its initial binding spot. Zoom-ins (black circles) highlight where and how the piston is initially bound. Black, magenta, blue, green and orange lines indicate the backbone of single and double stranded DNA. Short lines indicate individual bases of the DNA. Letters indicate the sequences of invader strands. Upon addition of the invader strands **(middle)** the piston is released from its initial binding spot by toehold-mediated strand displacement. The invader strands bind to their respective positions on the piston, defined by their sequences. The piston is then free to move back and forth along the direction of the filament **(bottom)**, indicated by the dashed black arrows.

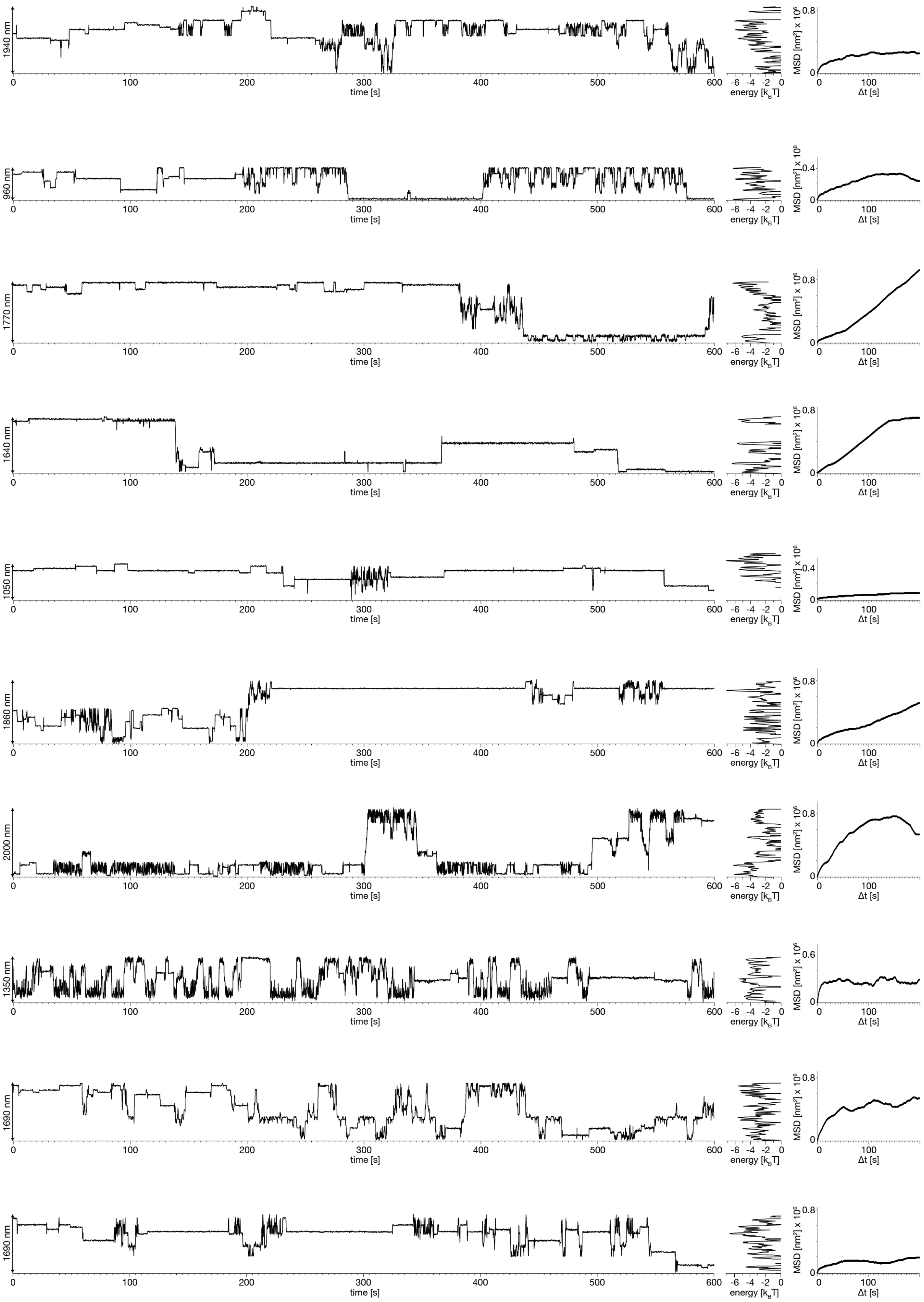

Supplementary Figure 25 | Left: Position-time traces of individual pistons (1-10) recorded at 20°C ambient temperature. Middle: Energy profiles computed from position probability distributions for the traces. Right: Mean square displacement (MSD) curves of the single particle traces.

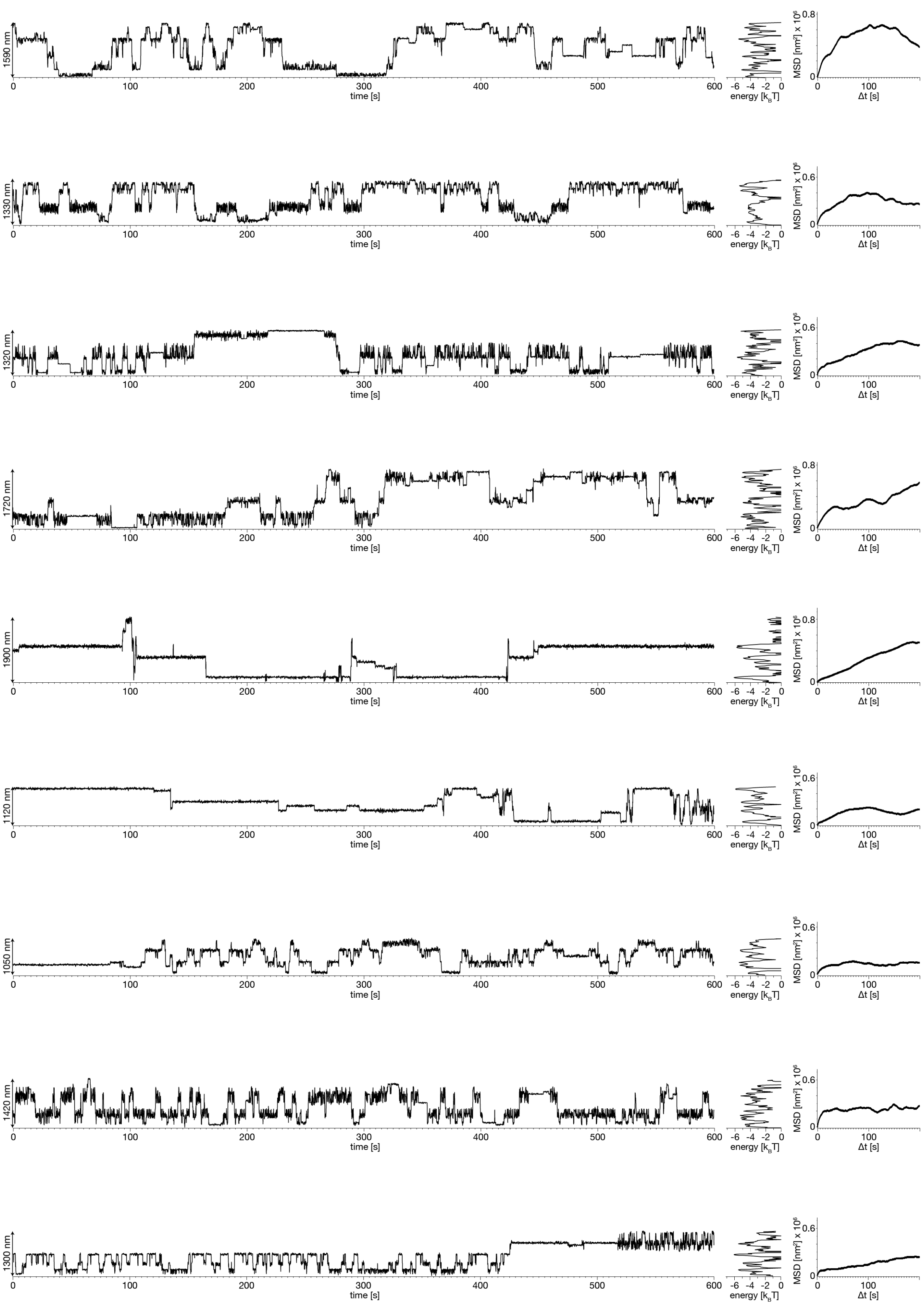

Supplementary Figure 26 | Left: Position-time traces of individual pistons (11-19) recorded at 20°C ambient temperature. Middle: Energy profiles computed from position probability distributions for the traces. Right: Mean square displacement (MSD) curves of the single particle traces.

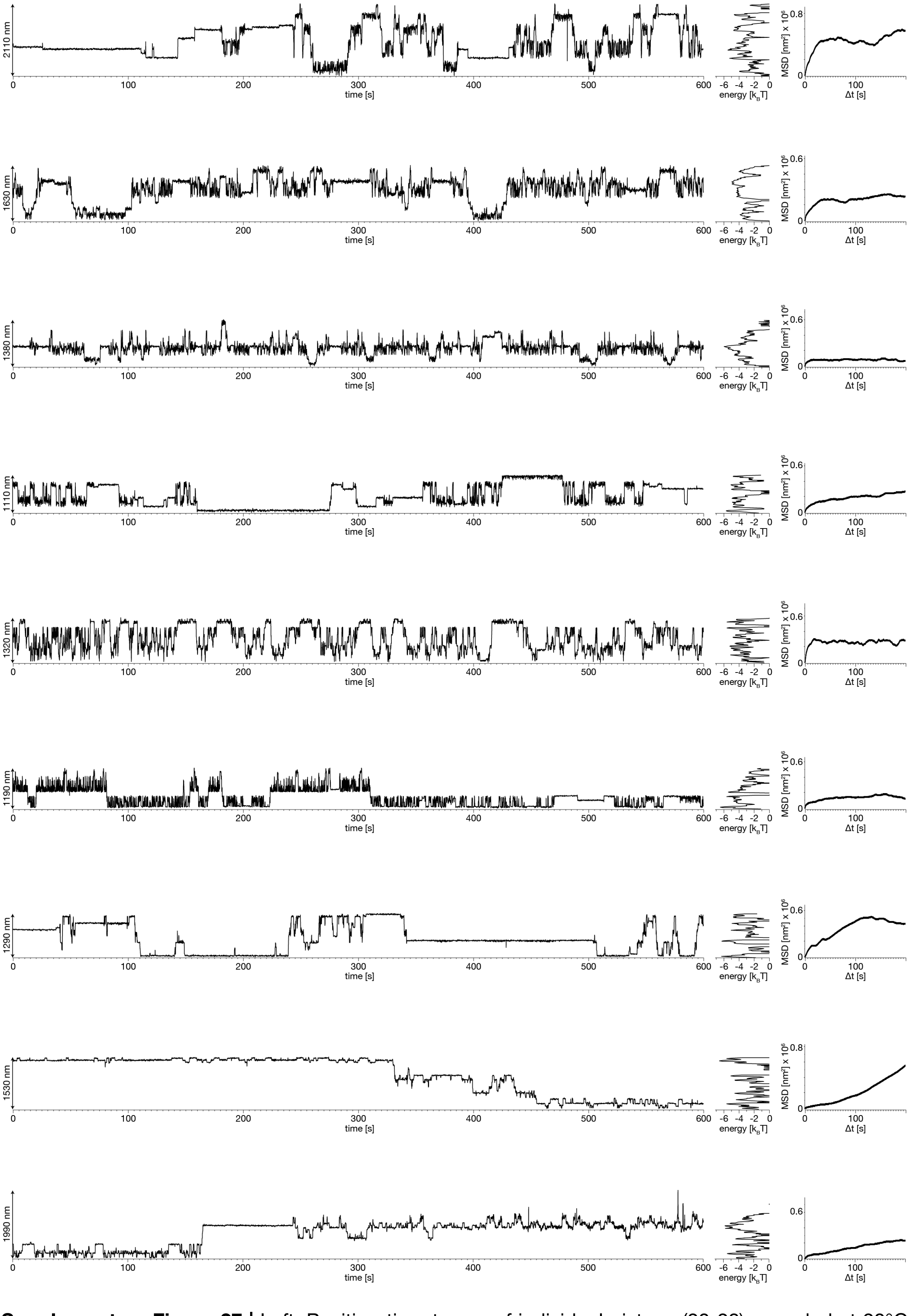

Supplementary Figure 27 | Left: Position-time traces of individual pistons (20-28) recorded at 20°C ambient temperature. Middle: Energy profiles computed from position probability distributions for the traces. Right: Mean square displacement (MSD) curves of the single particle traces.

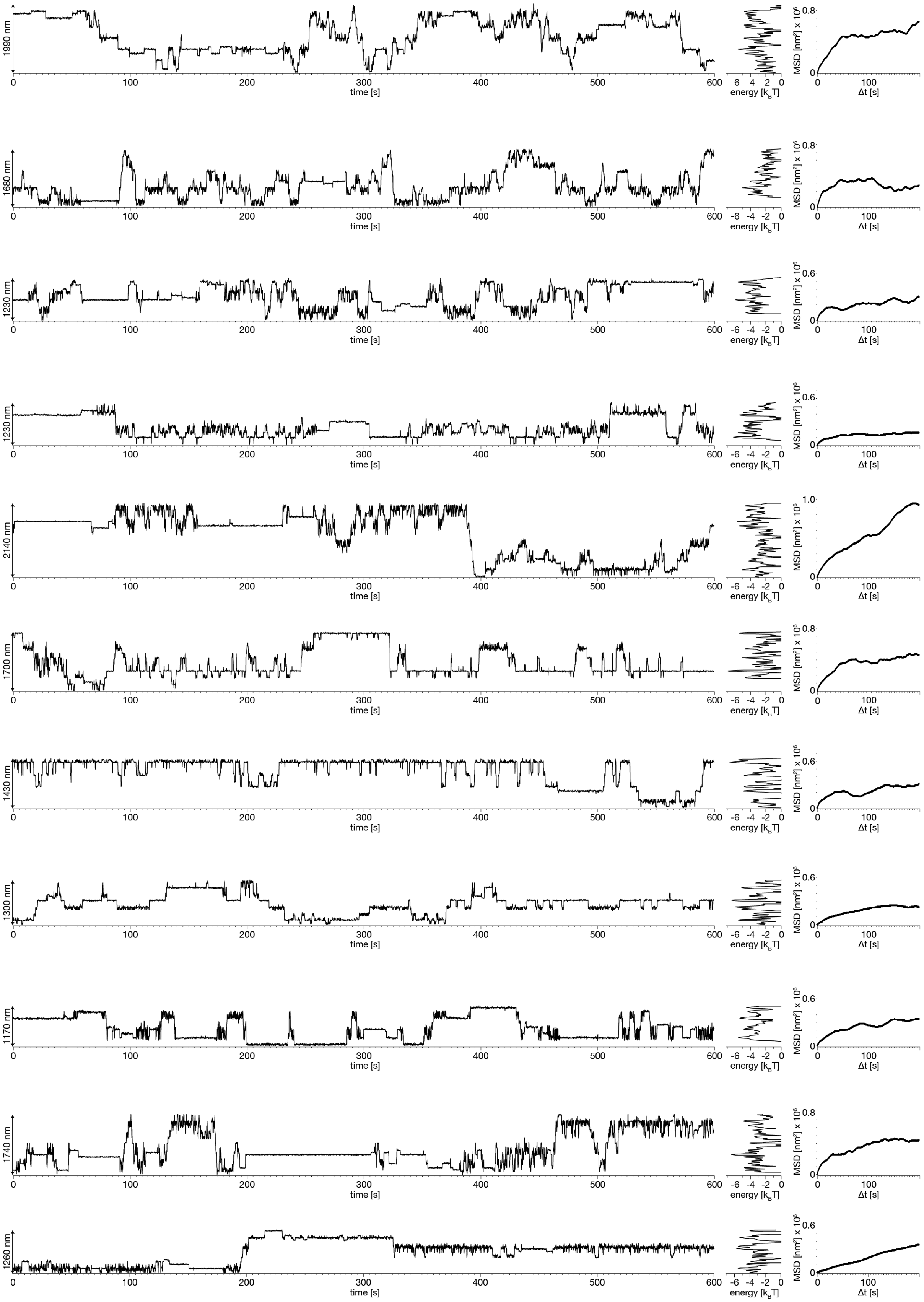

Supplementary Figure 28 | Left: Position-time traces of individual pistons (29-39) recorded at 25°C ambient temperature. Middle: Energy profiles computed from position probability distributions for the traces. Right: Mean square displacement (MSD) curves of the single particle traces.

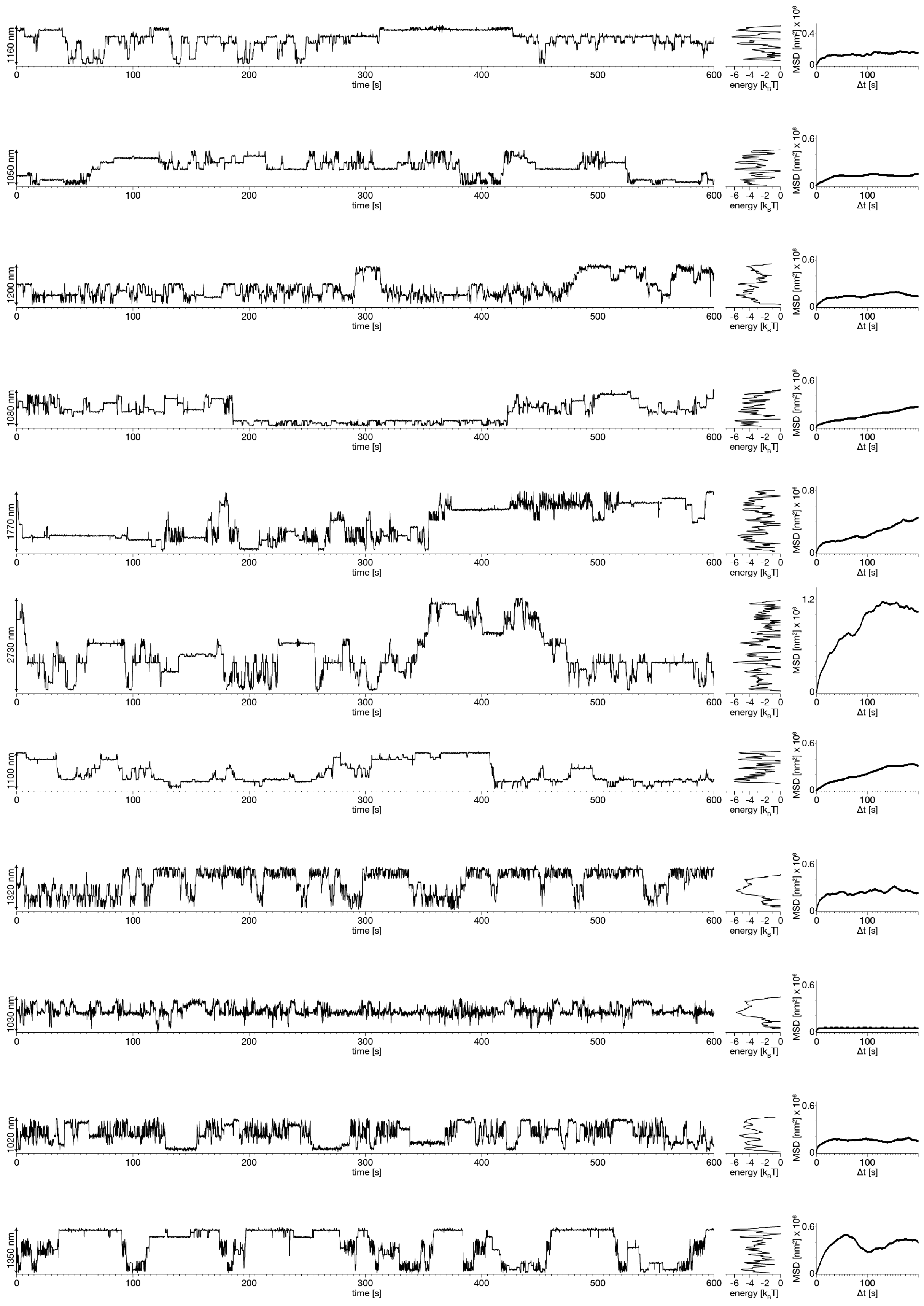

Supplementary Figure 29 | Left: Position-time traces of individual pistons (40-50) at 25°C ambient temperature. Middle: Energy profiles computed from position probability distributions for the traces. Right: Mean square displacement (MSD) curves of the single particle traces.

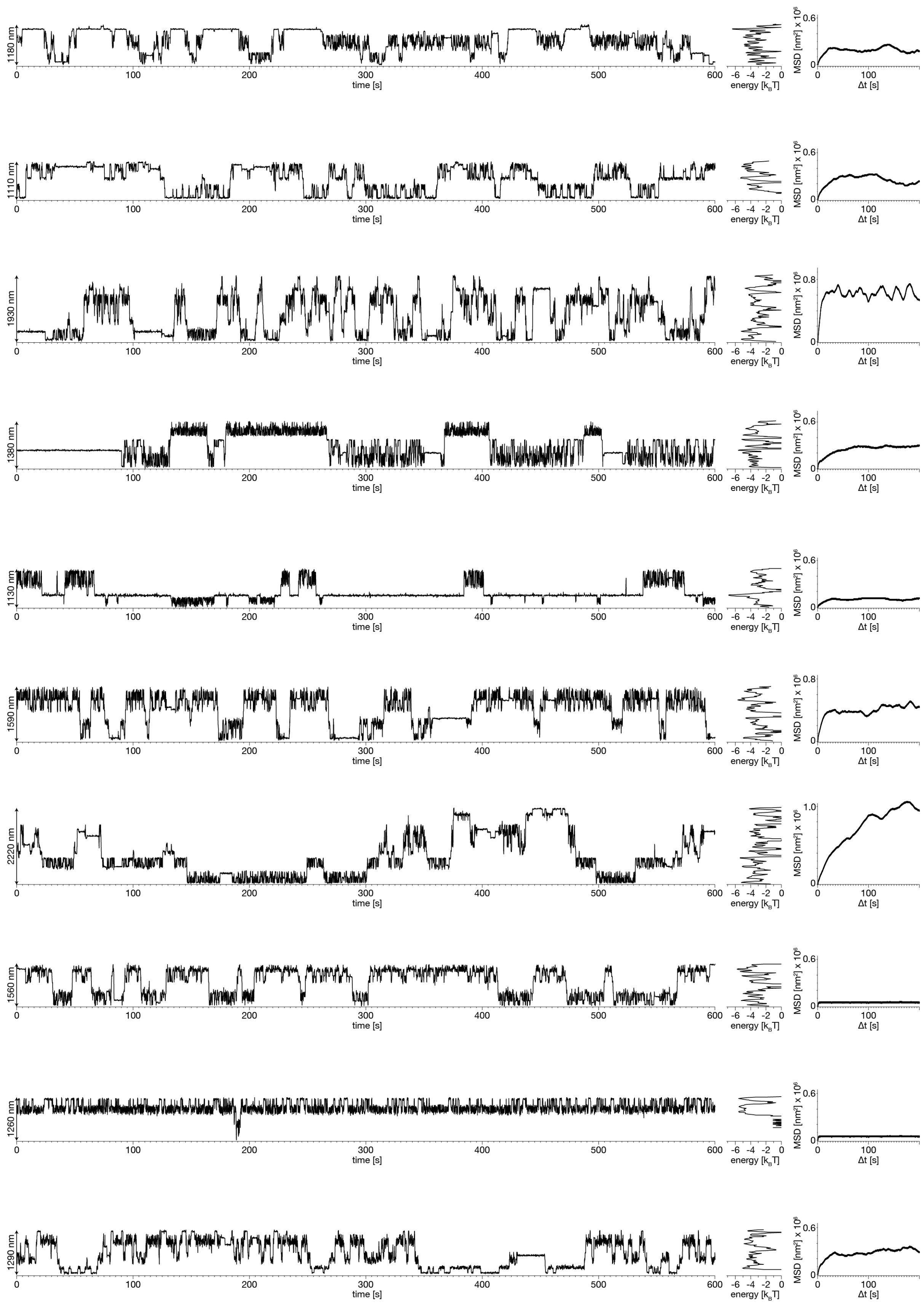

Supplementary Figure 30 | Left: Position-time traces of individual pistons (51-60) at 25°C ambient temperature. Middle: Energy profiles computed from position probability distributions for the traces. Right: Mean square displacement (MSD) curves of the single particle traces.

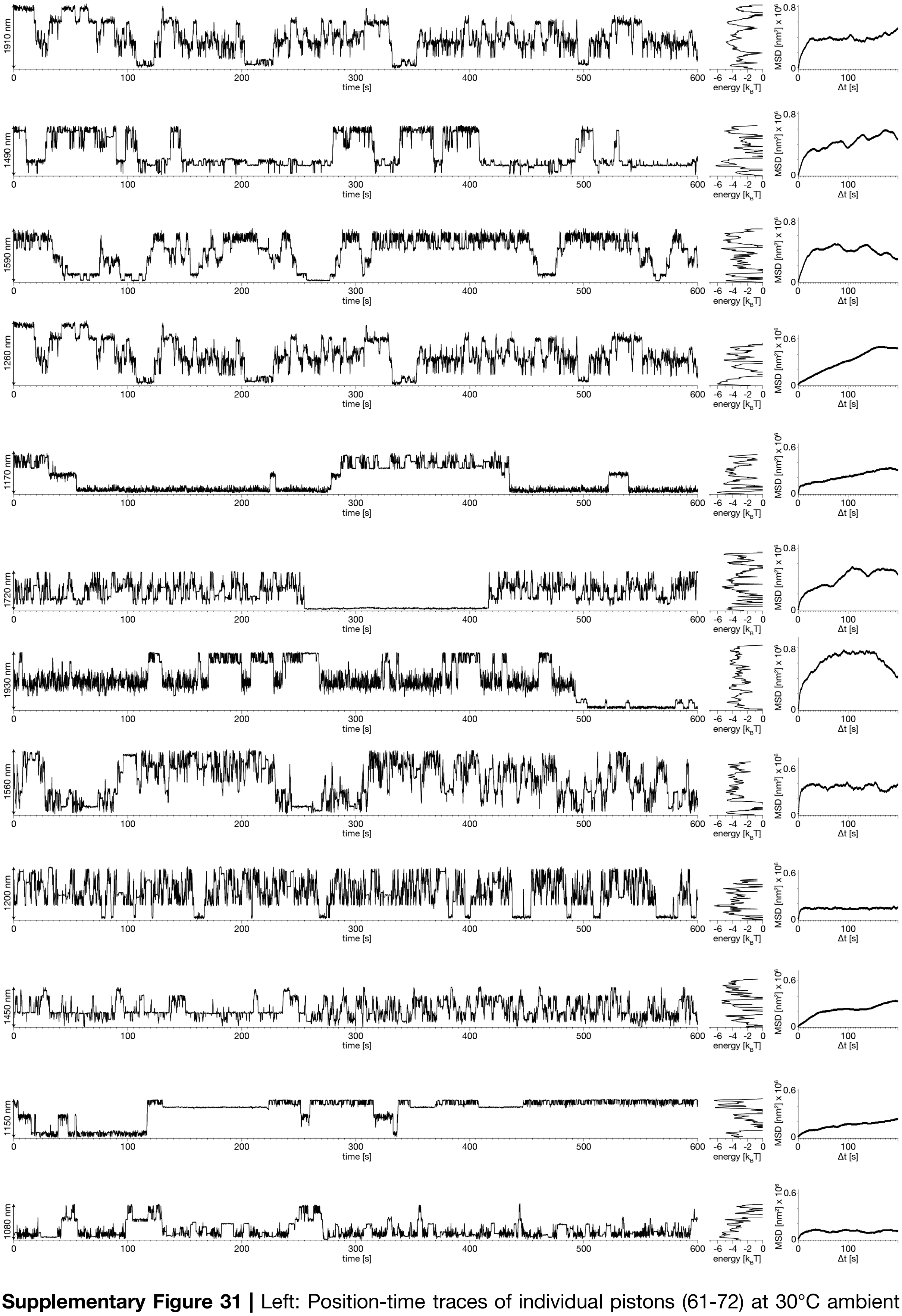

Supplementary Figure 31 | Left: Position-time traces of individual pistons (61-72) at 30°C ambient temperature. Middle: Energy profiles computed from position probability distributions for the traces. Right: Mean square displacement (MSD) curves of the single particle traces.

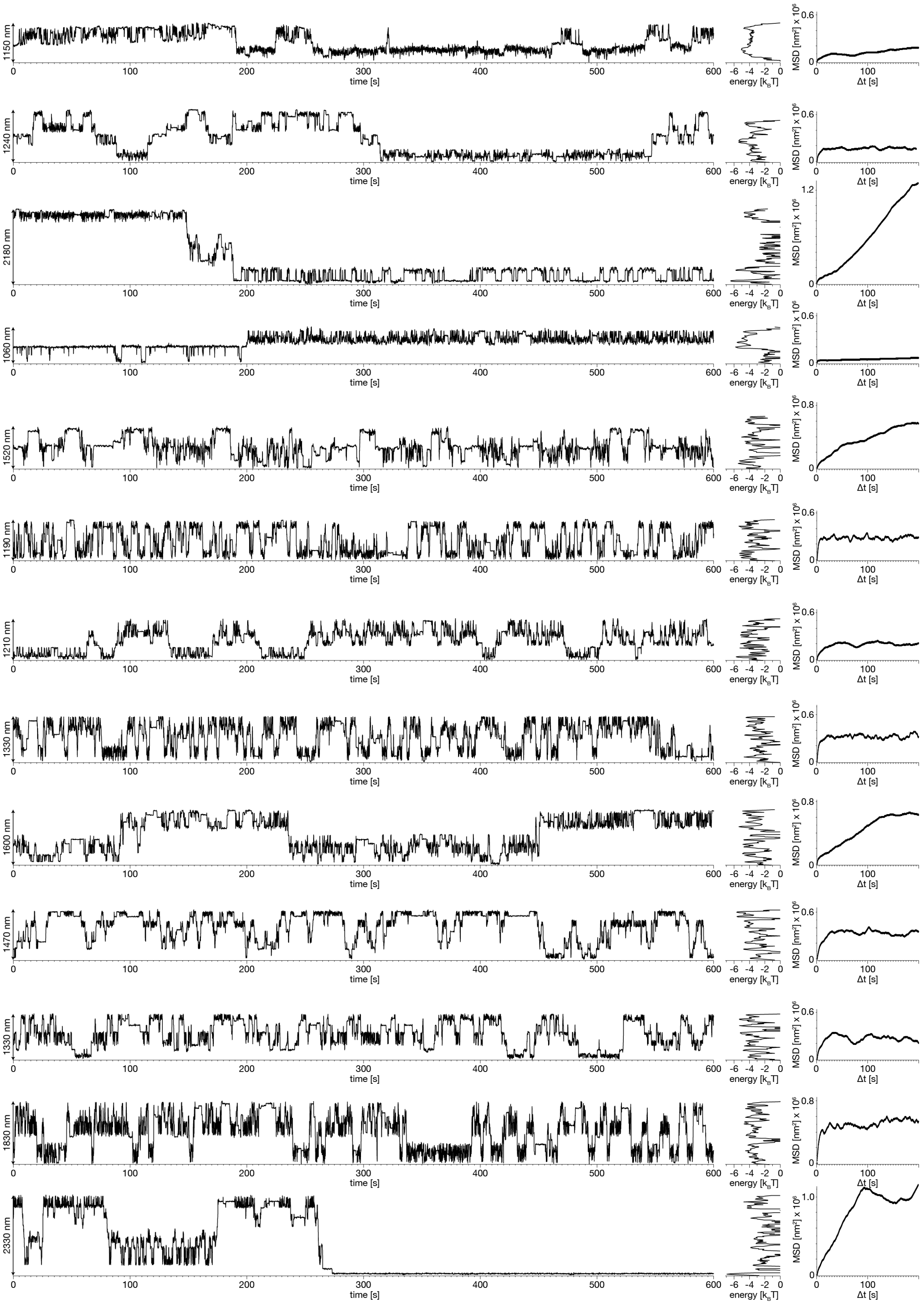

Supplementary Figure 32 | Left: Position-time traces of individual pistons (73-85) at 30°C ambient temperature. Middle: Energy profiles computed from position probability distributions for the traces. Right: Mean square displacement (MSD) curves of the single particle traces.

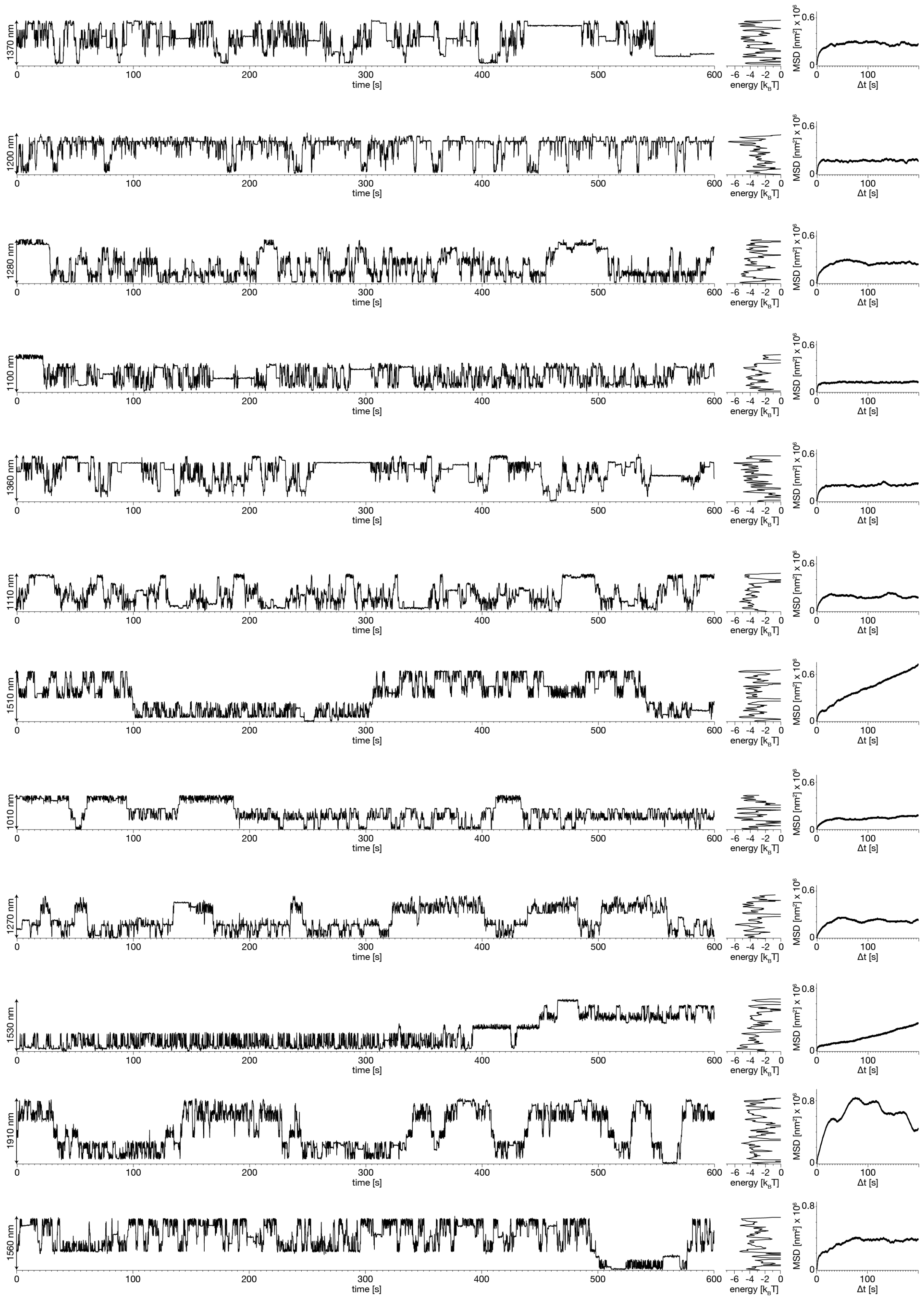

Supplementary Figure 33 | Left: Position-time traces of individual pistons (86-97) at 30°C ambient temperature. Middle: Energy profiles computed from position probability distributions for the traces. Right: Mean square displacement (MSD) curves of the single particle traces.

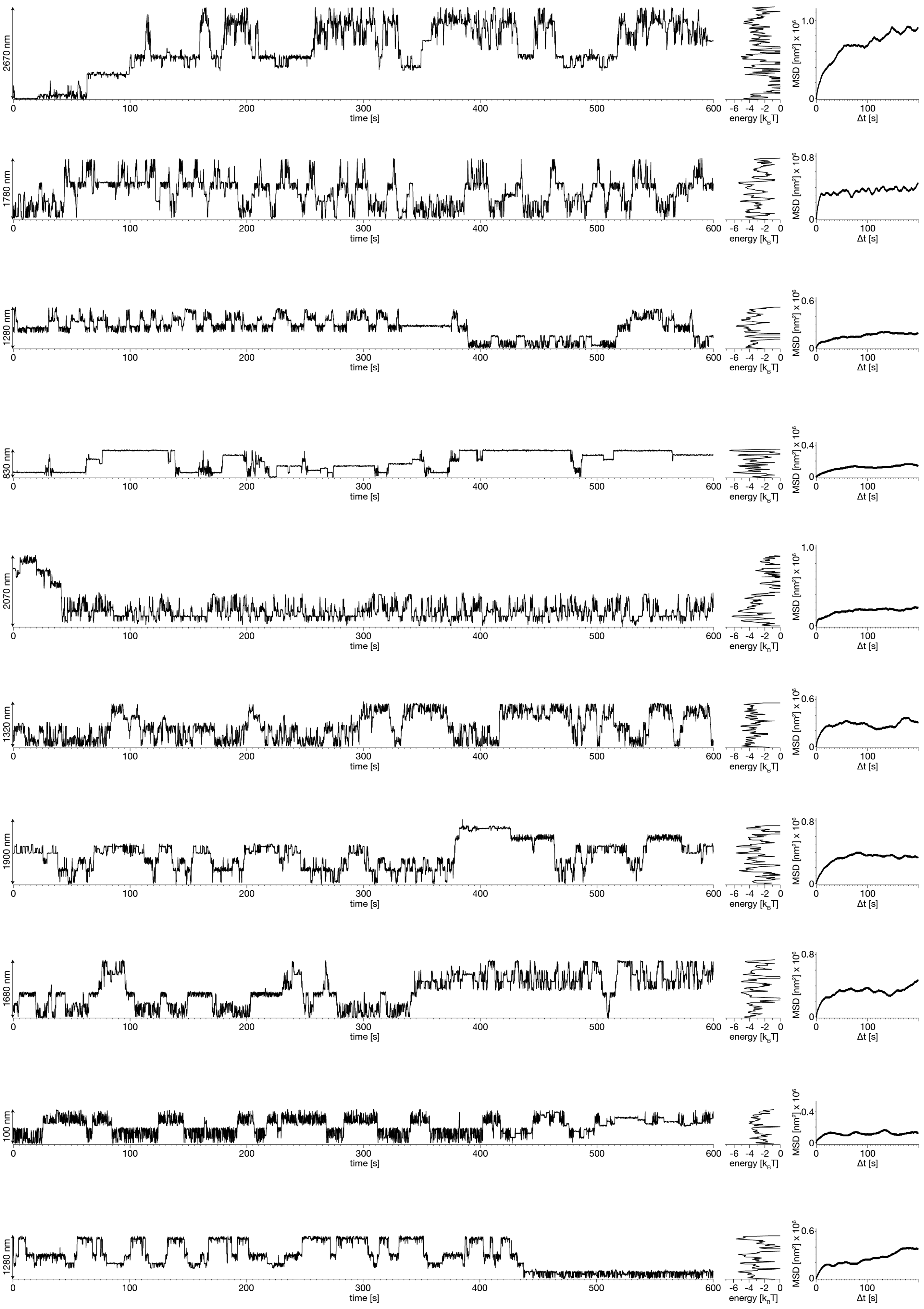

Supplementary Figure 34 | Left: Position-time traces of individual pistons (98-107) at 35°C ambient temperature. Middle: Energy profiles computed from position probability distributions for the traces. Right: Mean square displacement (MSD) curves of the single particle traces.

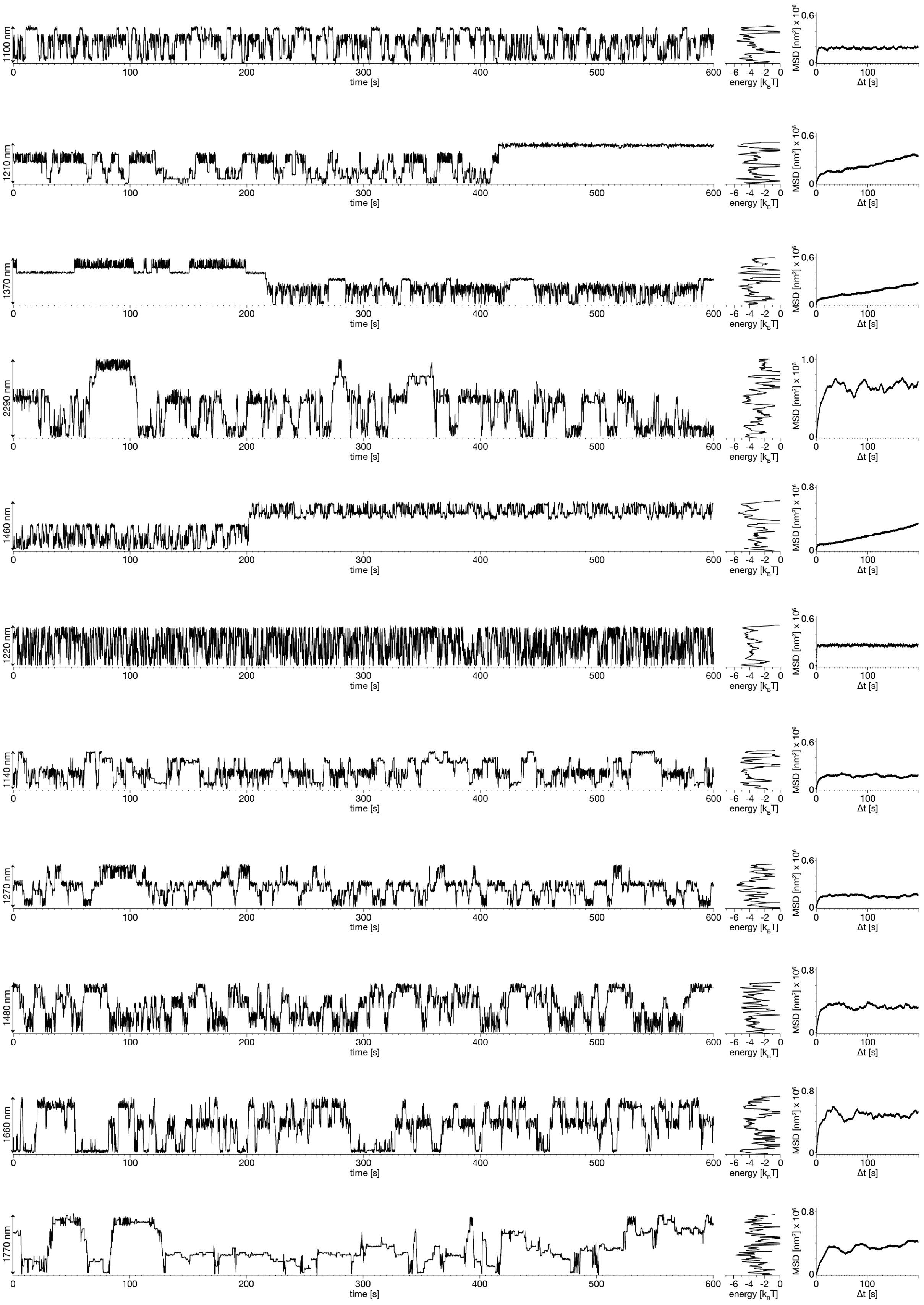

Supplementary Figure 35 | Left: Position-time traces of individual pistons (108-118) at 35°C ambient temperature. Middle: Energy profiles computed from position probability distributions for the traces. Right: Mean square displacement (MSD) curves of the single particle traces.

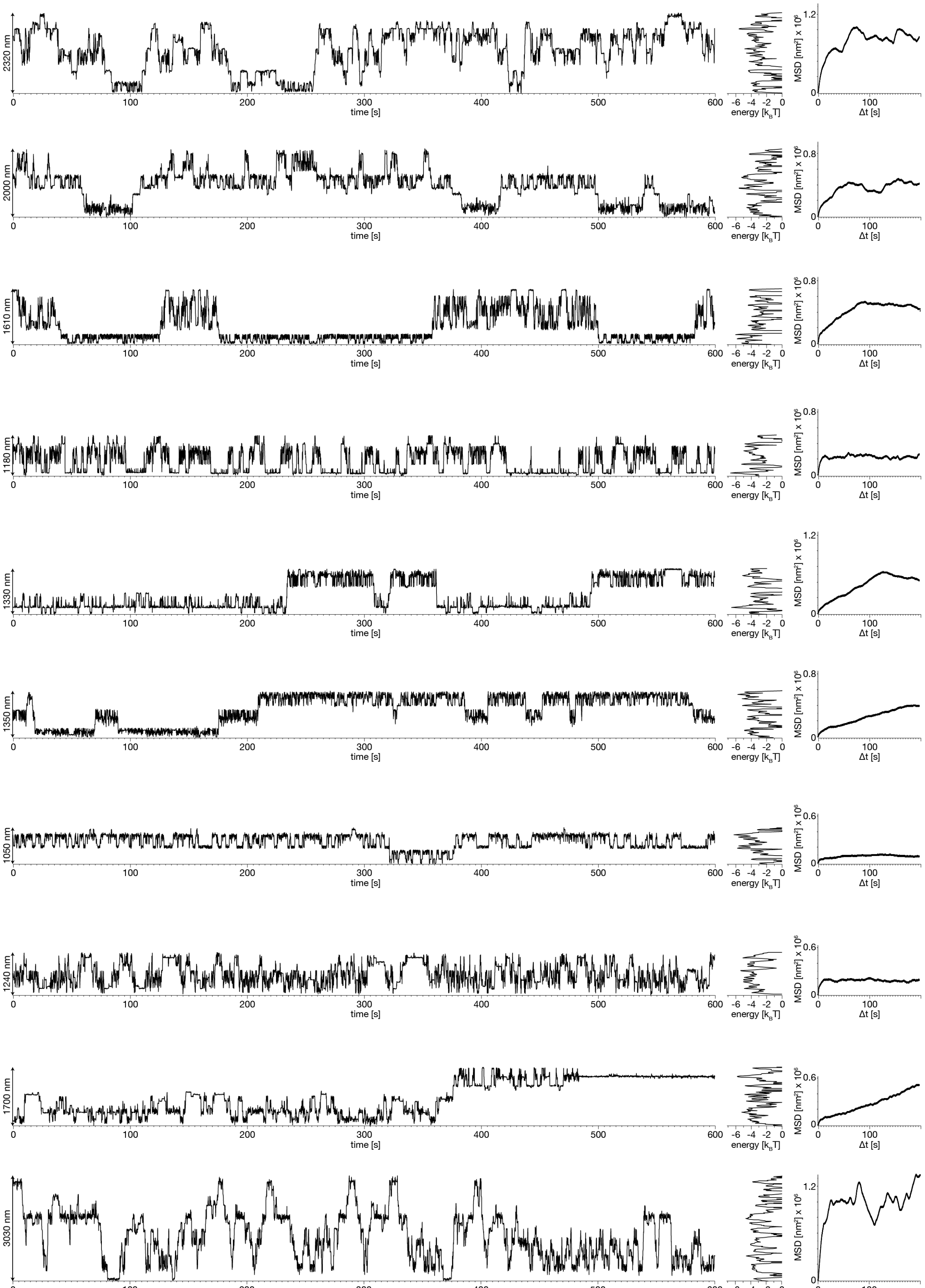

Supplementary Figure 36 | Left: Position-time traces of individual pistons (119-128) at 35°C ambient temperature. Middle: Energy profiles computed from position probability distributions for the traces. Right: Mean square displacement (MSD) curves of the single particle traces.

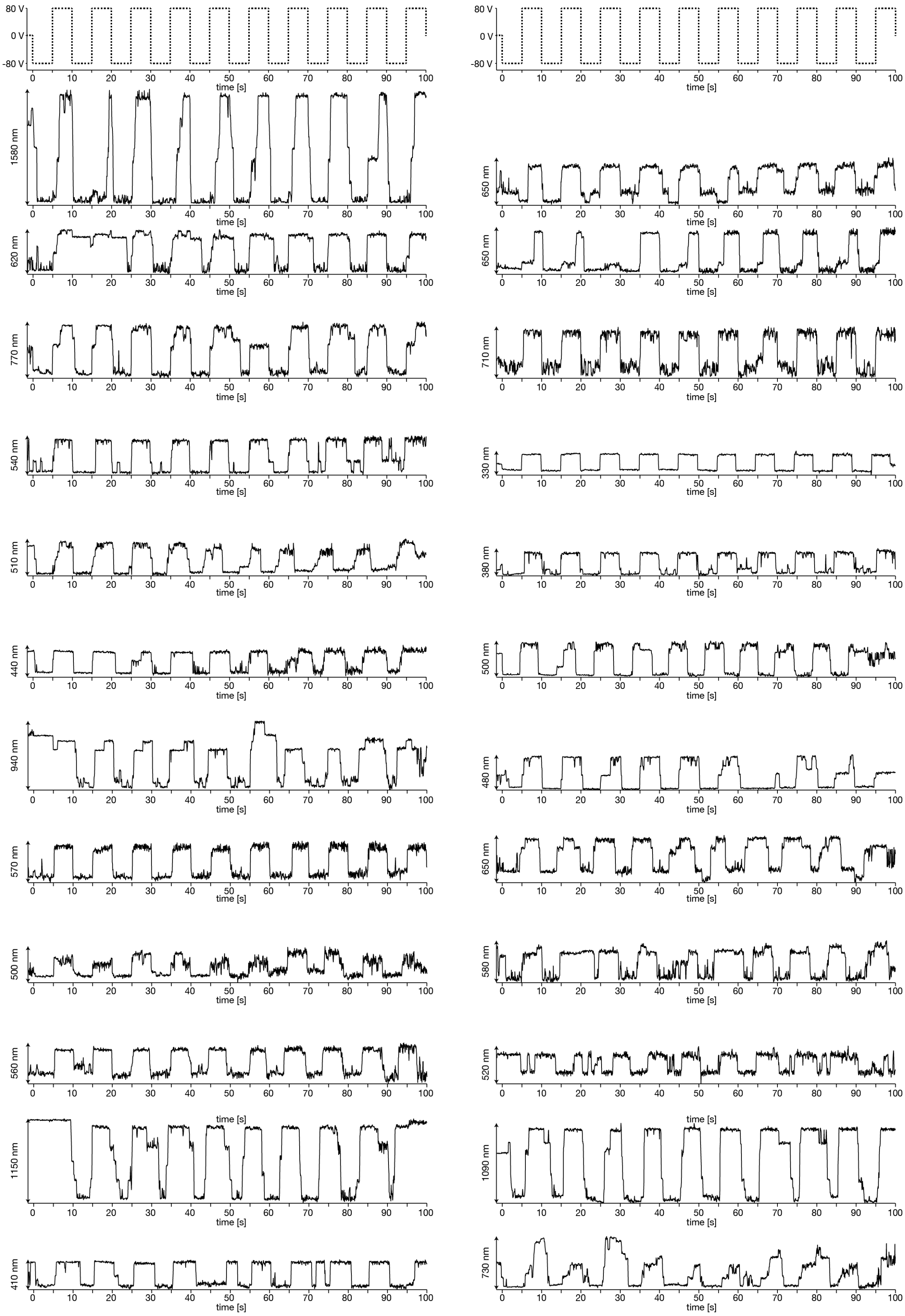

Supplementary Figure 37 | Driving the pistons with electric fields. Position-time traces of 24 pistons moving inside tunnels, with the tunnels oriented at angles between 0-50° relative to an applied electric field that was switched back and forth every 5 seconds (top row).

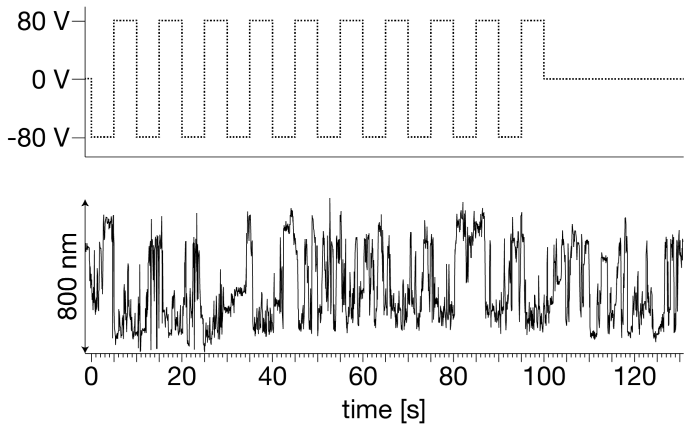

Supplementary Figure 38 | Single particle position-time trace of a piston inside of a tunnel which was oriented perpendicularly to an electric-field.

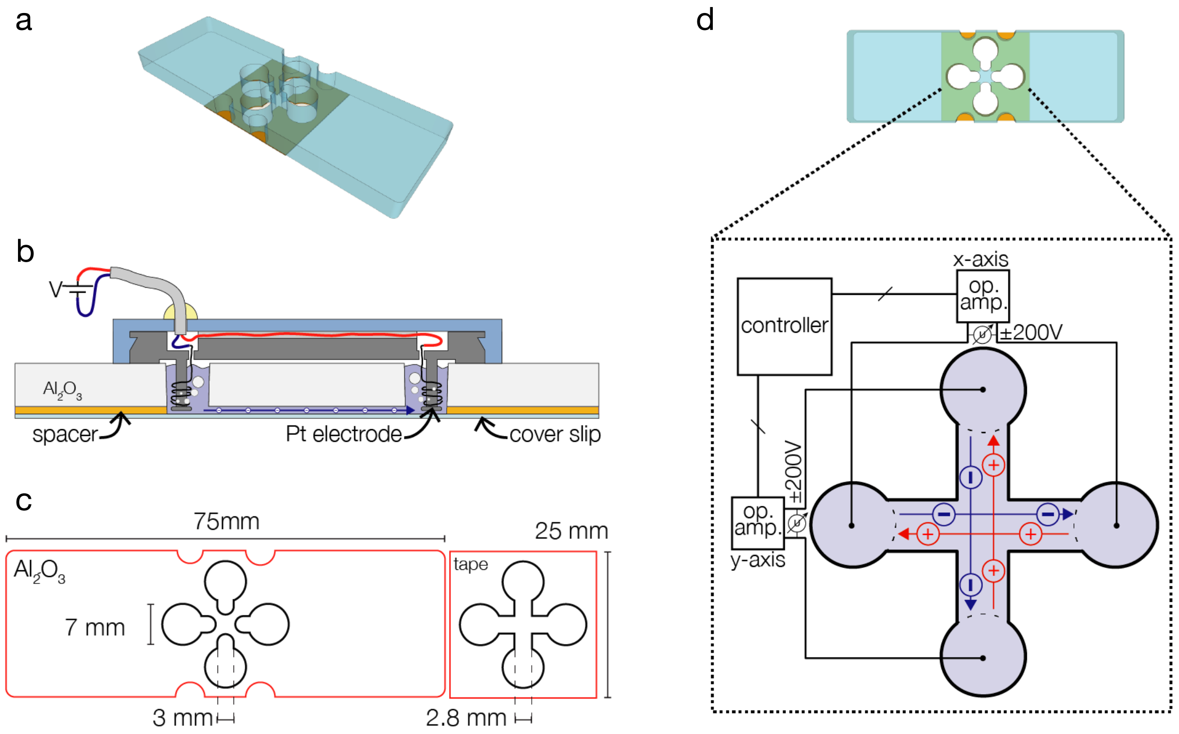

Supplementary Figure 39 | Electrode setup for the application of electric fields. (a) Illustration of assembled sample chamber in isometric view. (b) Cross section of sample chamber with inserted plug. For better visibility, material thickness not drawn to scale. (c) Dimensions of top component of sample chamber and adhesive tape. (d) Illustration of assembled sample chamber in top view and schematic of the resulting channel shape and electrode wiring.

Supplementary note 1

1.1. Langevin Simulations

The starting point of the theoretical description is the 1D Langevin equation for the diffusion of a particle in an external potential U(x), which reads

$$m\ddot{x}(t) = -\gamma\dot{x}(t) - \nabla U[x(t)] + F_R(t), \qquad \text{(S1)}$$

where m is the effective mass, γ is the friction coefficient, -$\nabla U[x(t)]$ is the force acting on the particle and $F_R(t)$ the random force which introduces the stochastic forces from the environment. We assume a stationary equilibrium Gaussian process with white noise, i.e. $< F_R(t) > = 0$ and $< F_R(t)F_R(0) > = B^2\,\delta(t)$, where $B = \sqrt{2k_B\,T\gamma}$.

A periodic sine or cosine potential is the simplest example of a periodic potential. However, the analysis of experimental data in fig. 4C suggests that the effective potential is much sharper than a sine or cosine potential. Hence, we choose a more general periodic potential landscape U of the form

$$U[x(t)] = U_B\left(1 - \sum_{n=0}^{N} e^{-\frac{(x(t)-\tilde{n}a)^2}{2b^2}}\right), \qquad \text{(S2)}$$

where a is the period, b is the width of the Gaussian potential well, U_B is the barrier height and $\tilde{n} = \frac{1+2n}{2}, n \in \mathbb{Z}$. In fig. S1, we show the potential for $U_B = 5\,k_BT$, b = 4 nm and a = 64 nm.

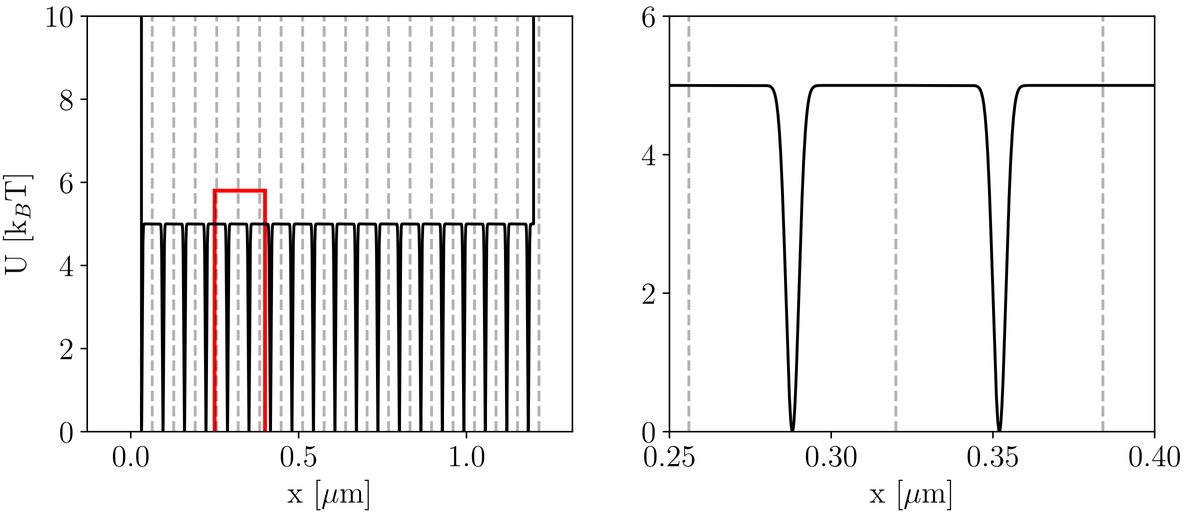

Supplementary Figure 40 | Left: Landscape of the periodic Gaussian potential in Eq. S2 with a period of a = 64 nm (see vertical dashed lines), a width of b = 4 nm and a barrier height of $U_B = 5\,k_BT$. Right: Close view of the potential in the area of the red inset in left.

We assume an overdamped motion, so that the Langevin equation in (S1) simplifies to

$$v(t) \equiv \dot{x}(t) = -\frac{1}{\gamma}\nabla U[x(t)] + \frac{1}{\gamma}F_R(t), \quad \text{(S3)}$$

The friction coefficient γ for the free diffusion case is estimated from the standard deviation of the averaged experimental velocity distribution in fig. 5E, i.e. $\sigma_{v,exp} \approx 0.59\,\mu m/s$. With $k_B T \approx 4.11 \cdot 10^{-21}$ J at 298 K we obtain:

$$\gamma = \frac{k_B T}{D} = \frac{2k_B T}{\sigma_{v,exp}^2 \Delta} \approx 1.39 \cdot 10^5 \,\frac{u}{fs}, \quad \text{(S4)}$$

where $\Delta = 0.1s$ is the experimental time step (frame rate of the detector). Using Einstein's relation for free diffusion, i.e. $\gamma = \frac{k_B T}{D}$, we have a diffusion coefficient of $D \approx 1.77 \cdot 10^{-2}\,\mu m^2/s$. With the experimentally found friction coefficient, we can estimate the inertial time scale $\tau_m = m/\gamma$. With m = 2 MDa, we have $\tau_m \approx 14\,fs$ for room temperature. This small value suggests that our overdamped assumption in Eq. S3 is valid.

We simulate trajectories by numerically solving Eq. S3 using the fourth-order Runge-Kutta method with a time step δt. We adjust the potential $U[x(t)]$ to experimental conditions. At positions x = 0 μm and x = 1.2 μm, we add infinitely high potential barriers to the periodic potential landscape (see fig. S 39), which approximately correspond to the length of a filament that is closed at both ends. These boundaries are reflective, which is a realistic assumption for the movement of a piston in the filament.

We sample time steps for the random force F_R by computing Gaussian white noise with zero mean and variance

$$\sigma_F^2 = \langle F_R(0)F_R(0) \rangle, \quad \text{(S5)}$$

$$= B^2/\delta t, \quad \text{(S6)}$$

$$= 2k_B\, T\, \gamma/\delta t. \quad \text{(S7)}$$

The mean and variance expression correspond to the choice of random force in the Langevin equation in Eq. S1. At each numerical time step F_R^i, we draw a random number from a Gaussian distribution $\mathcal{N}$ with zero mean and a standard deviation of σ_F in Eq. S7, i.e. $F_R^i \sim \mathcal{N}(0, \sigma_F)$. We simulate trajectories with a time resolution of δt =10 μs and a total length of 600 s. The simulated trajectories x_i are discretized and used to calculate the discrete velocity

$$v_i(t) = \frac{x_i(t+\Delta/2) - x_i(t-\Delta/2)}{\Delta}, \quad \text{(S8)}$$

with $\Delta = 0.1\,s$ being the experimental time resolution. In fig. S 40, we show exemplary simulated, discretized trajectories with the potential landscape in Eq. S2 for different barrier heights. We observe that the standard deviation of the velocity probability distribution depends on the barrier height choice. This suggests that the non-Gaussian behavior found in the experimental data arises from the summation of piston traces in periodic potentials with different barrier heights.

For the simulation results we show in fig. 5E, we simulated 100 of these trajectories with randomly chosen potential barrier heights from a uniform distribution in the range between $U_B = 1\,k_B\,T$ and $U_B = 10\,k_B\,T$, a potential period a = 64 nm and a barrier width of b = 4 nm. We kept the potential landscape homogeneous for each simulation, meaning constant barrier height over time and space. Afterwards, we computed the discrete velocity distributions with Eq. S8 to obtain the result in fig. 5E.

The choice of the individual barrier heights was based on estimates from the experimentally found potential landscapes. However, these profiles suffer from limited sampling, and no exact combination of heights could be determined, so random heights from a uniform distribution were chosen. Therefore, we assume that a specific "optimal" combination of barrier heights can completely reproduce the experimental distribution.

1.2. Adding Localization Noise to the Simulations

The simulations based on the Langevin miss an important experimental ingredient. The detection method based on fluorescence videos using super-resolution centroid tracking is subject to a limited resolution. We tracked pistons before adding invader strands. These pistons should be stationary and provide a good basis for estimating the spatial resolution. The spatial resolution can be considered as natural localization noise added to the actual piston position. Regarding the simulation setup, such localization noise of the piston's position can be modeled by adding a Gaussian random variable $x_{loc}(t)$ with zero mean and adjustable standard deviation to the discrete position (*2*). The model prediction for the experimentally measured position reads as $x_{exp}(i\Delta) = x_{sim}(i\Delta) + x_{loc}(i\Delta)$. For the simulation results we show in fig. 5D and fig. S 39, we added localization noise x_{loc} sampled from a Gaussian distribution with zero mean and a standard deviation of $\sigma_{loc} = 5$ nm, i.e. $x_{loc}(i\Delta) \sim \mathcal{N}(0, \sigma_{loc})$, to the discretized trajectories.

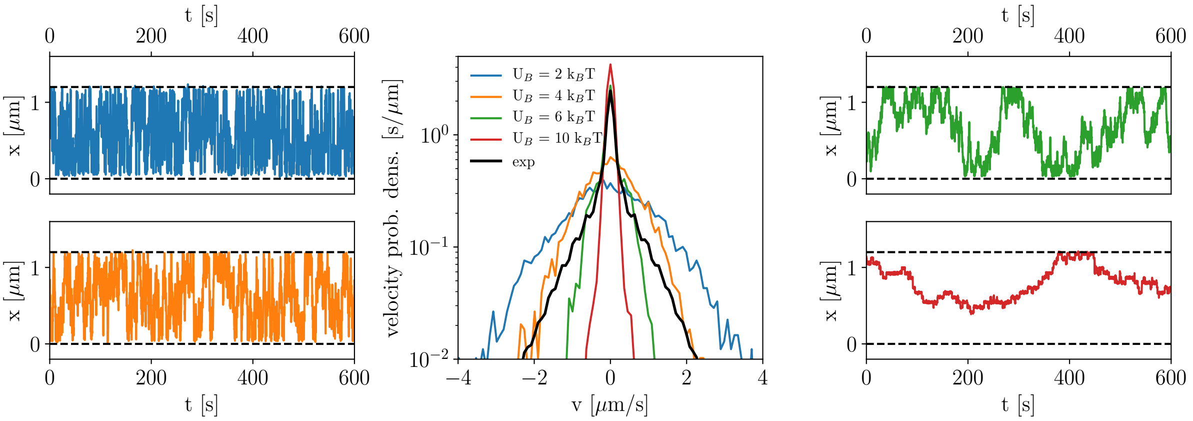

Supplementary Figure 41 | Examples of overdamped Langevin simulations in a periodic Gaussian potential (see Eq. S2) with simulation resolution $\delta t = 10\ \mu s$ and discretized to a time resolution of $\Delta = 0.1$ s, with a period of a = 64 nm, a width of b = 4 nm and varying barrier height U_B (denoted by different colors). For each simulation, the potential landscape was kept homogeneous, meaning with constant barrier height over time and space. Localization noise $x_{loc}(i\Delta)$ sampled from a Gaussian distribution with zero mean and a standard deviation of $\sigma_{loc} = 5$ nm was added to the discretized trajectory positions. Here we used k_BT for 298 K and $\gamma = 1.39 \cdot 10^5\ \frac{u}{fs}$ (estimated by Eq. S4). Middle: Calculated discrete velocity probability distributions of the individual simulations (colored, Eq. S8) in comparison with the averaged velocity distribution of the experimental trajectories (black).

Supplementary References

1. S. M. Douglas *et al.*, Rapid prototyping of 3D DNA-origami shapes with caDNAno. *Nucleic acids research* **37**, 5001-5006 (2009).
2. B. G. Mitterwallner, C. Schreiber, J. O. Daldrop, J. O. Radler, R. R. Netz, Non-Markovian data-driven modeling of single-cell motility. *Phys Rev E* **101**, 032408 (2020).
