# Supplementary material for: A synthetic tubular molecular transport system
Source: Nat Commun. 2021 Jul 20;12:4393. doi: 10.1038/s41467-021-24675-8 (PMC8292359; doi:10.1038/s41467-021-24675-8)
Supplement: Supplementary file 2 — Reporting Summary [file 41467_2021_24675_MOESM2_ESM.pdf]

nature portfolio

Corresponding author(s): Hendrik Dietz
Last updated by author(s): Jun 24, 2021

Reporting Summary

Nature Portfolio wishes to improve the reproducibility of the work that we publish. This form provides structure for consistency and transparency in reporting. For further information on Nature Portfolio policies, see our Editorial Policies and the Editorial Policy Checklist.

Statistics

For all statistical analyses, confirm that the following items are present in the figure legend, table legend, main text, or Methods section.

n/a	Confirmed	
☐	☒	The exact sample size (n) for each experimental group/condition, given as a discrete number and unit of measurement
☒	☐	A statement on whether measurements were taken from distinct samples or whether the same sample was measured repeatedly
☒	☐	The statistical test(s) used AND whether they are one- or two-sided<br>*Only common tests should be described solely by name; describe more complex techniques in the Methods section.*
☒	☐	A description of all covariates tested
☒	☐	A description of any assumptions or corrections, such as tests of normality and adjustment for multiple comparisons
☐	☒	A full description of the statistical parameters including central tendency (e.g. means) or other basic estimates (e.g. regression coefficient) AND variation (e.g. standard deviation) or associated estimates of uncertainty (e.g. confidence intervals)
☒	☐	For null hypothesis testing, the test statistic (e.g. F, t, r) with confidence intervals, effect sizes, degrees of freedom and P value noted<br>*Give P values as exact values whenever suitable.*
☒	☐	For Bayesian analysis, information on the choice of priors and Markov chain Monte Carlo settings
☒	☐	For hierarchical and complex designs, identification of the appropriate level for tests and full reporting of outcomes
☒	☐	Estimates of effect sizes (e.g. Cohen's d, Pearson's r), indicating how they were calculated

Our web collection on statistics for biologists contains articles on many of the points above.

Software and code

Policy information about availability of computer code

Data collection	AMT 600, Image Capture Engine Software Version 600.307a; SerialEM 3.5.6 and 3.8.0 beta; LabView version 15.0f2 (self written code inside LabView); Typhoon FLA 9500 Control Software, version 1.1, build 1.1.0.187; PIMikroMove version 2.31.2.0; caDNAno v0.2
Data analysis	IgorPro version 6.37 (custom written scripts inside IgorPro for bulk data analysis); Relion 2.0 up to 3.0.8 beta; Cryolo 1.0 up to 1.5; ImageJ version 2.1.0/1.53c, NanoJ-Core drift correction plugin in ImageJ, Mosaic Particle Tracker plugin in ImageJ; NanoDrop 8000 software, Photoshop CS6; MotionCorr 2

For manuscripts utilizing custom algorithms or software that are central to the research but not yet described in published literature, software must be made available to editors and reviewers. We strongly encourage code deposition in a community repository (e.g. GitHub). See the Nature Portfolio guidelines for submitting code & software for further information.

Data

Policy information about availability of data

All manuscripts must include a data availability statement. This statement should provide the following information, where applicable:

- Accession codes, unique identifiers, or web links for publicly available datasets
- A description of any restrictions on data availability
- For clinical datasets or third party data, please ensure that the statement adheres to our policy

All data are available from the corresponding author upon reasonable request.

Field-specific reporting

Please select the one below that is the best fit for your research. If you are not sure, read the appropriate sections before making your selection.

☒ Life sciences ☐ Behavioural & social sciences ☐ Ecological, evolutionary & environmental sciences

For a reference copy of the document with all sections, see nature.com/documents/nr-reporting-summary-flat.pdf

Life sciences study design

All studies must disclose on these points even when the disclosure is negative.

Sample size	The focus of this study is the proof of concept (proof of existence) of fast 1D travel over micrometer distances. We quantified the diffusive behavior of individual particles if the travel ranges exceeded 1 µm. These exclusion criteria were not pre-established. The total number of particles analyzed was > 100.
Data exclusions	Fluorescence measurements: Particles with travel ranges below 1 µm were not considered for further analysis (see point above). Tunnel filaments with more than 1 moving particle were excluded from centroid tracking analysis, because correct particle assignment and tracking was not possible. For 2D class averaging of EM images we used 2D classification with Relion averaging routine and manually excluded particles in classes with obvious structural defects. These exclusion criteria were not pre-established.
Replication	We performed multiple assembly replicates, that is, replicates of the entire self-assembly procedure to create long 1D filaments with mobile units, as self-assembled from stock solutions of the 4 distinct building blocks as explained in figure 1 (or variants of those blocks). We also performed multiple technical replicates, that is, for example, repeated fluorescence measurements of the same filament-mobile unit assembly on different microscopy slides, or for transmission electron microscopy. The overall number of assembly replicates in this study is 4 for samples used in free diffusion measurements and 2 for the electric field driven measurements with the design variants shown in the paper. The number of technical replicates for each assembly replicate was typically at least three. Following the exact sample preparation steps outlined in the supplement of this study, all attempts at replication were successful.
Randomization	Randomization is not relevant for this study because no statistical tests are needed in this proof of concept type study. We analyzed all acquired particles with travel ranges exceeding 1 µm.
Blinding	Blinding is not relevant for this study because particles acquired at different temperatures were all treated with the same automated analysis tools. Other treatment and control groups are not relevant and were not considered.

Reporting for specific materials, systems and methods

We require information from authors about some types of materials, experimental systems and methods used in many studies. Here, indicate whether each material, system or method listed is relevant to your study. If you are not sure if a list item applies to your research, read the appropriate section before selecting a response.

Materials & experimental systems

n/a	Involved in the study
☒	☐ Antibodies
☒	☐ Eukaryotic cell lines
☒	☐ Palaeontology and archaeology
☒	☐ Animals and other organisms
☒	☐ Human research participants
☒	☐ Clinical data
☒	☐ Dual use research of concern

Methods

n/a	Involved in the study
☒	☐ ChIP-seq
☒	☐ Flow cytometry
☒	☐ MRI-based neuroimaging
