# Supplementary material for: A synthetic tubular molecular transport system
Source: Nat Commun. 2021 Jul 20;12:4393. doi: 10.1038/s41467-021-24675-8 (PMC8292359; doi:10.1038/s41467-021-24675-8)
Supplement: Supplementary file 4 — Description of Additional Supplementary Files [file 41467_2021_24675_MOESM4_ESM.pdf]

**Title: Supplementary Data File 1**

**Description:** DNA sequences needed to fold the piston, named “potential_dye_positon_1-15”, “end_1-7”, “core_1-8”. DNA sequences needed to release the bound piston from the barrel, named “invader_1-4”. Scaffold sequence needed to fold the piston, named “scaffold”.

**Title: Supplementary Data File 2**

**Description:** DNA sequences needed to fold the barrel, named “core_1-282”, “piston_bind_1-4”, “no_piston_bind_1-8”, “Cy5_oligo_1-10”, “anchor_1-2”, “biotin_oligo”. DNA sequences needed to close the barrel, named “close_1-8”. DNA sequences needed to polymerize the barrel, named “first_1-27”, “second_1-69”. Scaffold sequences needed to fold the barrel, named “scaffold_1”, “scaffold_2”

**Title: Supplementary Data File 3**

**Description:** DNA sequences needed to fold the capping object 1, named “core_1-86”, “end1_1-68”, “core2_1-8”, “sticky_1-21”, “end2_1-30”. Scaffold sequences needed to fold the capping object 1, named “scaffold”.

**Title: Supplementary Data File 4**

**Description:** DNA sequences needed to fold the capping object 2, named “core_1-95”, “end1_1-30”, “core2_1-9”, “sticky_1-19”, “end2_1-66”. Scaffold sequences needed to fold the capping object 2, named “scaffold”.

**Title: Supplementary Software File 1**

**Description:** Simulation code, named “lang_sim_overdamped_delta_pot.py”, to compute velocity distributions without different barrier heights and localization-noise.

**Title: Supplementary Software File 2**

**Description:** Code, named “sim_langevin_filaments.py”, used to compute velocity distributions with different barrier heights and localization-noise.

**Title: Supplementary Software File 3**

**Description:** Code, used within IgorPro, for batch processing of single-particle piston traces.
function rotategraph (): function to rotate piston traces.
function generateallvelocs (): function to generate velocities from single-particle piston traces.
function msd (): function to generate mean square displacements of pistons.
function correlateprobs (): function to generate autocorrelations of probability distributions of piston positions.
function movewavestoroot (): function to move data around different folders.
function histo (): function to generate histograms of positions of pistons.
function partofwave () and function partofwave2 (): functions to generate positions of pistons of the first and second half of the piston traces.
function killgarbage (): function to delete data.
function msd_sat (): function to generate mean square displacements of parts of pistons traces.
function max_displacement (): function to generate maximum displacements of pistons.

**Title: Supplementary Movie 1**
**Description:** Exemplary fluorescence microscopy movie with released pistons (merged channels). Real-time. 50 x 50 µm.

**Title: Supplementary Movie 2**
**Description:** Exemplary fluorescence microscopy movie with released pistons (merged channels). Real-time. Each pixel is 104 nm.

**Title: Supplementary Movie 3**
**Description:** Exemplary fluorescence microscopy movie with released piston (merged channels). Real-time. Each pixel is 104 nm.

**Title: Supplementary Movie 4**
**Description:** Longest range: Exemplary fluorescence microscopy movie with released piston (merged channels). Real-time. Each pixel is 104 nm.

**Title: Supplementary Movie 5**

**Description:** Fastest particle: Exemplary fluorescence microscopy movie with released piston (merged channels). Real-time. Each pixel is 104 nm.

**Title: Supplementary Movie 6**

**Description:** Electric-field driven motion: Exemplary fluorescence microscopy movie with released piston (merged channels). Real-time. Each pixel is 130 nm.

**Title: Supplementary Movie 7**

**Description:** Electric-field driven motion: Exemplary fluorescence microscopy movie with released pistons (merged channels). Real-time. Each pixel is 130 nm.
